# Anti-lymphangiogenesis for boosting drug accumulation in tumors

**DOI:** 10.1038/s41392-024-01794-4

**Published:** 2024-04-15

**Authors:** Chunling Wang, Junchao Xu, Xiaoyu Cheng, Ge Sun, Fenfen Li, Guangjun Nie, Yinlong Zhang

**Affiliations:** 1 CAS Key Laboratory for Biomedical Effects of Nanomaterials & Nanosafety, CAS Center for Excellence in Nanoscience, National Center for Nanoscience and Technology, Beijing, 100190 China; 2 Sino-Danish College, University of Chinese Academy of Sciences, Beijing, 100190 China; 3 Sino-Danish Center for Education and Research, Beijing, 100190 China; 4 Department of Bioengineering, University of Pennsylvania, Philadelphia, PA 19104 USA; 5 School of Nanoscience and Engineering, University of Chinese Academy of Sciences, Beijing, 100049 China

**Keywords:** Cancer microenvironment, Cancer therapy

## Abstract

The inadequate tumor accumulation of anti-cancer agents is a major shortcoming of current therapeutic drugs and remains an even more significant concern in the clinical prospects for nanomedicines. Various strategies aiming at regulating the intratumoral permeability of therapeutic drugs have been explored in preclinical studies, with a primary focus on vascular regulation and stromal reduction. However, these methods may trigger or facilitate tumor metastasis as a tradeoff. Therefore, there is an urgent need for innovative strategies that boost intratumoral drug accumulation without compromising treatment outcomes. As another important factor affecting drug tumor accumulation besides vasculature and stroma, the impact of tumor-associated lymphatic vessels (LVs) has not been widely considered. In the current research, we verified that anlotinib, a tyrosine kinase inhibitor with anti-lymphangiogenesis activity, and SAR131675, a selective VEGFR-3 inhibitor, effectively decreased the density of tumor lymphatic vessels in mouse cancer models, further enhancing drug accumulation in tumor tissue. By combining anlotinib with therapeutic drugs, including doxorubicin (Dox), liposomal doxorubicin (Lip-Dox), and anti-PD-L1 antibody, we observed improved anti-tumor efficacy in comparison with monotherapy regimens. Meanwhile, this strategy significantly reduced tumor metastasis and elicited stronger anti-tumor immune responses. Our work describes a new, clinically transferrable approach to augmenting intratumoral drug accumulation, which shows great potential to address the current, unsatisfactory efficacies of therapeutic drugs without introducing metastatic risk.

## Introduction

The accumulation of therapeutic agents within tumor tissues is a crucial determinant of their anti-tumor effects. While for small molecular drugs, their indiscriminate distribution across various organs not only limits their anti-cancer efficacy but also results in severe toxicities. Nanomedicines, which can benefit from the enhanced permeability and retention (EPR) effect, have long been regarded as a promising strategy to improve the therapeutic efficacy of free chemotherapeutics and overcoming the clinical obstacles associated with their systemic toxicity due to the lack of specificity.^1,2^ In preclinical researches, countless nanomedicines have been extensively developed, with several of them achieving clinical approval for cancer therapy.^3,4^ However, recently, a growing number of clinical trials have demonstrated that nanomedicines do not exhibit superior anti-cancer efficacy.^5–8^ Moreover, studies have revealed that the actual delivery efficiency of nanoparticles to solid tumors is low.^9^ Although nanomedicines exhibit advantages in some aspects of cancer therapy, e.g., reduced systemic toxicity, improved pharmacokinetic properties, controllable drug release, and protective effects for protein- and nucleic acid-based drugs, their unsatisfactory anti-cancer efficacy greatly impedes their clinical usage.^1^ The compromised intratumoral accumulation and penetration of nanomedicines have been attributed to the complexity and heterogeneity of the tumor microenvironment (TME), which varies across different disease stages or among individuals.^10^ Taking measures to augment intratumoral drug accumulation is critical for the successful application of nanomedicines in the clinic, and is vital to enhance the anti-cancer efficacy of conventional therapeutics.^11^

The heterogeneity of tumor blood vessels is the largest factor interfering with drug accumulation and has been a major focus of recent research. Due to the compression or obstruction by the dense tumor extracellular matrix (ECM), the rapidly proliferating cancer cells, and abnormal blood coagulation, the permeability of tumor vessels is greatly hampered.^12–14^ Based on this understanding of tumor biology, various strategies have been developed, including the inhibition of ECM production, the disruption of the tumor vessel barrier, and the elimination of thrombi, to improve drug accumulation in tumor tissue.^15–19^ In addition, the ability of nanoparticles to penetrate into tumor tissue relies not only on the spaces between endothelial cells but also on multiple mechanisms, such as active trans-endothelial transport and induction of endothelial leakiness (NanoEL).^20–22^ Developing nanoparticles that exhibit the NanoEL effect or possess transcytosis capabilities represents an emerging approach to enhancing the accessibility of therapeutic drugs to tumors. However, these strategies targeting tumor blood vessels or stroma also raise potential concern about increasing the risk of metastasis.^23–25^

Emerging evidence indicates the presence of functional lymphatic vessels (LVs) within TME, which exert a significant impact on tumor progression.^26,27^ Vessel endothelial growth factor C (VEGF-C) or VEGF-D secreted by tumor cells or immune cells promotes lymphangiogenesis and LV remodeling through the VEGF-C/D-VEGFR-3 axis.^26–28^ Tumor lymphatic metastasis is one of the leading causes of mortality, and high levels of LV within a tumor are associated with poor outcomes in several types of cancer.^29,30^ Therefore, anti-lymphangiogenesis is a promising strategy for cancer therapy.^31^ Most importantly, several studies have shown that intratumoral macromolecules or nanoparticles can be drained from tumor tissue to draining lymph nodes (LNs) via LVs,^32–34^ indicating the involvement of tumor-associated LVs in substance transport from tumors to draining LNs. With the aforementioned knowledge of LVs in mind, we hypothesize that anti-lymphangiogenesis could serve as an efficacious strategy to improve intratumoral drug accumulation and simultaneously reduce tumor metastasis.

**Fig. 1:**
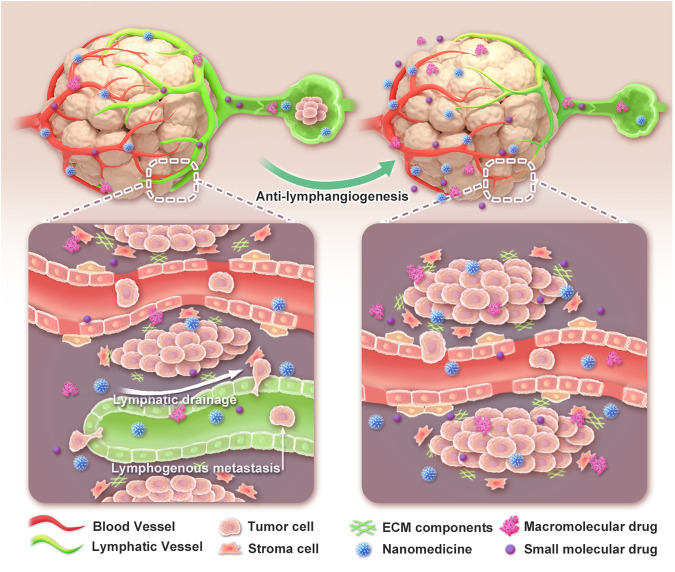
Schematic illustration of the intratumoral accumulation of therapeutic drugs after anti-tumor lymphangiogenesis. With cancer progression, lymphangiogenesis and lymphatic enlargement occur in and around the primary tumor. These processes not only diminish the intratumoral accumulation of therapeutic drugs but also facilitate metastasis. By inhibiting lymphangiogenesis, increased amounts of drugs can be trapped in the tumor tissue, and lymphatic metastasis is reduced

In the current study, we employed anlotinib, a multi-target tyrosine kinase inhibitor (TKI) with anti-lymphangiogenesis ability,^35^ and SAR131675, a selective VEGFR-3 TKI with potent anti-lymphangiogenesis activity,^36^ to demonstrate that the anti-lymphangiogenesis strategy can augment the accumulation of nanoparticles, macromolecules, and free drugs in tumor tissue (Fig. 1). Systemic administration of anlotinib significantly improved the anti-tumor efficacy of both liposomal doxorubicin (Lip-Dox) and free doxorubicin (Dox). More significantly, anti-lymphangiogenesis inhibited tumor colonization in draining LNs and prevented distant metastasis. Anlotinib treatment also increased the infiltration of CD4^+^ and CD8^+^ T cells in tumors and exhibited a net immunostimulatory effect. Combined with its ability to enhance intratumoral accumulation, anlotinib synergistically improved the anti-cancer efficacy of the anti-PD-L1 antibody. Our research offers a proof-of-concept combined strategy of anti-lymphangiogenesis with therapeutic drugs, showing great potential for cancer therapy with reduced metastatic risk.

## Results

### Anti-lymphangiogenesis activity of anlotinib and SAR131675

**Fig. 2:**
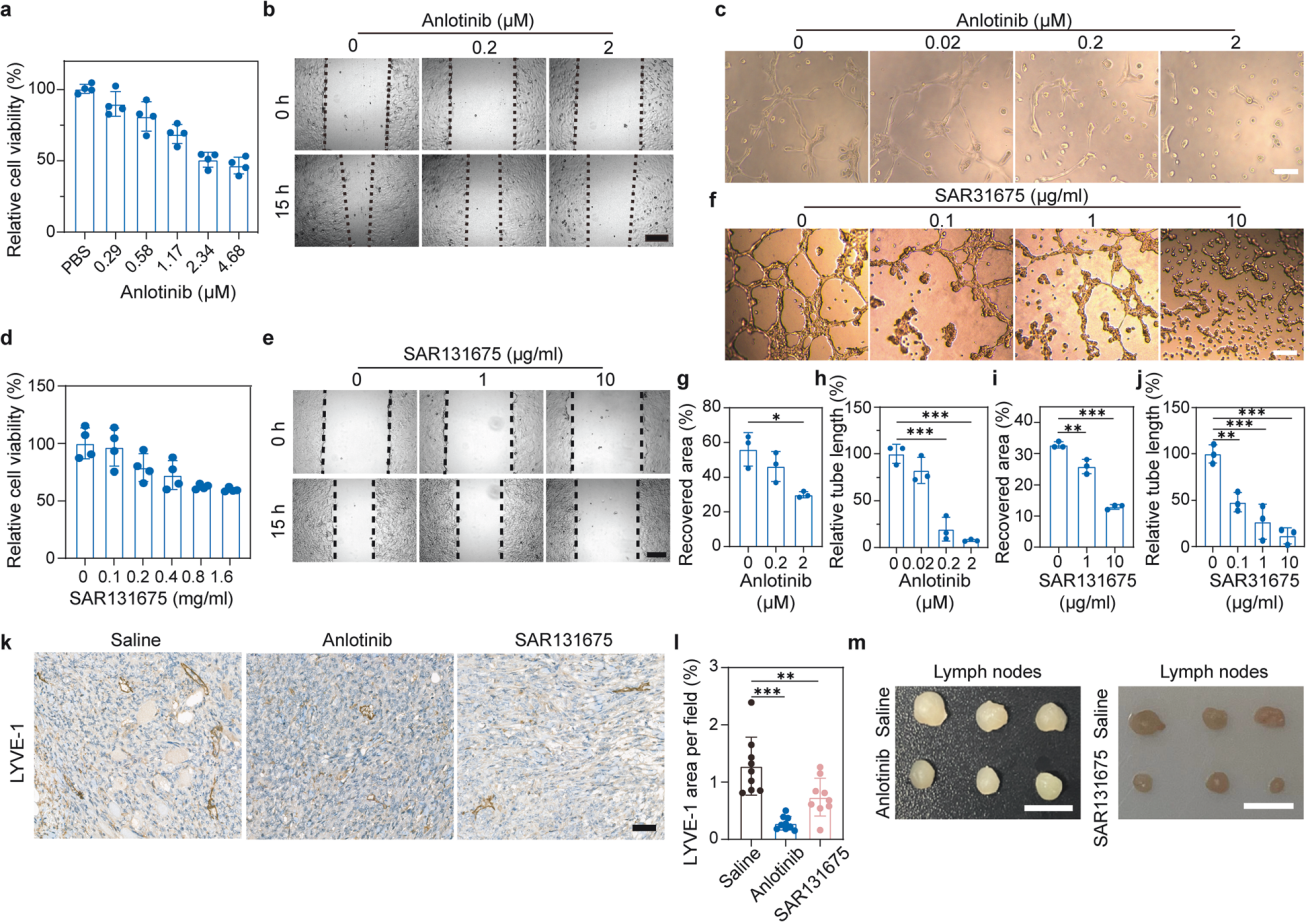
Anti-lymphangiogenesis activity of anlotinib and SAR131675 both in vitro and in vivo. **a**–**c** Anlotinib inhibited the proliferation (**a**), migration (**b**), and tubule network formation (**c**) of hLECs (*n* = 4). Scale bar, 200 μm in (**b**) and 1000 μm in (**c**). **d**–**f** SAR131675 inhibited the proliferation (**d**), migration (**e**), and tubule network formation (**f**) of hLECs (*n* = 4). Scale bar, 200 μm in (**e**) and 1000 μm in (**f**). **g** Quantification of the recovered area by hLECs migration as shown in (**b**) (*n* = 3). **h** Quantification of the recovered area by hLECs migration as shown in and (**e**) (*n* = 3). **i** Quantification of tube lengths as shown in (**c**) (*n* = 3). **j** Quantification of tube lengths as shown in (**f**) (*n* = 3). **k** Immunohistochemistry staining for LVs (LYVE-1, brown) in 4T1 tumor sections from mice treated with saline, anlotinib or SAR131675. Scale bar, 40 μm. **l** Quantification of tumor LV density (*n* = 9; images were from three mice per group). **m** Typical images of inguinal LNs from different groups. Scale bar, 0.5 cm. The data are presented as the mean ± s.d. *p < 0.05; **p < 0.01; ***p < 0.001

The process of tumor lymphangiogenesis includes three key steps: proliferation, migration, and tubule network formation of lymphatic endothelial cells (LECs).^37^ The survival, proliferation, and migration of LECs depend on the VEGF-C/VEGFR-3 axis, which is known to induce phosphorylation in downstream molecules such as Erk and Akt.^27^ We first investigated the phosphorylation of VEGFR-3, Akt, and Erk of human LECs (hLECs) after treatment with anlotinib and SAR131675 by Western Blots. As shown in Supplementary Fig. S1, both anlotinib and SAR131675 significantly inhibited VEGF-C-induced phosphorylation of VEGFR-3, Erk, and Akt. Next, we investigated the anti-lymphangiogenesis activities of anlotinib and SAR131675 by evaluating their impacts on cell viability, wound healing, and tubule formation. To evaluate the impacts of anlotinib on the proliferation of LECs, we incubated hLECs in the presence of various dosages of anlotinib for 24 h. As measured using the cell counting kit-8 assay (CCK-8), anlotinib suppressed the proliferation of hLECs in a dose-dependent manner (Fig. 2a). Additionally, results of wound healing assay demonstrated that anlotinib could suppress the migration of hLECs (Fig. 2b, g), and tubule network formation of hLECs was interrupted after treatment with anlotinib (Fig. 2c, i). Similarly, SAR131675 exhibited an inhibitory effect on hELCs proliferation dose-dependently (Fig. 2d), and disturbed migration (Fig. 2e, h) and tubule network formation (Fig. 2f, j) of hLECs. Collectively, these in vitro results demonstrate that both anlotinib and SAR131675 can inhibit lymphangiogenesis by inhibiting the proliferation, migration, and tubule network formation of hLECs. We next tested the in vivo anti-tumor lymphangiogenesis activity of the two drugs. Mice bearing 4T1 tumors received saline, anlotinib or SAR131675 treatment for 10 consecutive days. Immunohistochemical staining for the LV marker, LYVE-1, in tumor sections, showed that the density of LVs obviously decreased after anlotinib or SAR131675 treatment (Fig. 2k, l). Additionally, the anti-lymphangiogenesis activity of anlotinib was further confirmed via immunofluorescence staining of 4T1 and CT26 tumor tissues, as evidenced by the decreased expression of LYVE-1 (Supplementary Fig. S2). Western blot analysis revealed that anlotinib and SAR131675 treatment consistently decreased the expression of LYVE-1 in the 4T1 tumors (Supplementary Fig. S3). In addition, the volume of tumor-draining LNs in the anlotinib or SAR131675 treatment groups was markedly smaller than those in the saline group (Fig. 2m).

### Drainage function of tumor-associated LVs

**Fig. 3:**
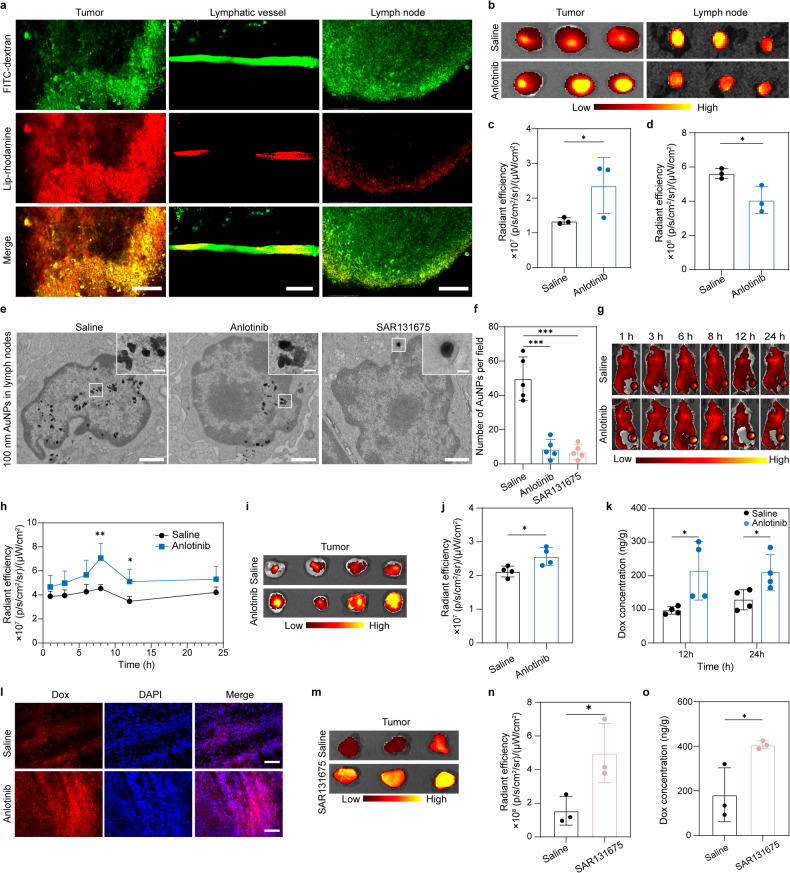
Decreased drainage of nanoparticles from tumor to draining LNs, and enhanced intratumoral accumulation of nanoparticles or free Dox after lymphangiogenesis inhibition. **a** Representative multiphoton laser scanning microscopy images of the drainage of tumor-associated LVs for Lip-Rhodamine. Scale bar, 100 μm in the right and left panels and 50 μm in the middle panels. **b** Ex vivo fluorescence images of tumors and draining LNs 8 h after intratumoral injection of Lip-Cy5.5 in 4T1 tumor-bearing mice. **c**, **d** Quantitative analysis of the fluorescence intensities in the tumors (**c**) and draining LNs (**d**) 8 h post-injection (*n* = 3). **e**, **f** After treatment with saline, anlotinib or SAR131675, the mice were intratumorally administered with 100 nm AuNPs. Representative TEM images of inguinal LNs are shown (**e**), and the number of AuNPs per field was quantified (**f**) (*n* = 5). Scale bar, 1 μm. The upper panels show enlarged images of the indicated areas of interest. Scale bar, 100 nm. **g**–**j** Anlotinib treatment enhanced the intratumoral accumulation of nanoparticles. After treatment with saline or anlotinib, the mice were intravenously administered 40 nm fluorescent microspheres. Representative in vivo fluorescence images at different time points are shown (**g**). Tumors were circled with black lines, and their average fluorescence intensities were quantified (**h**) (*n* = 4). Tumors were excised 24 h post-administration for ex vivo imaging (**i**), and the average fluorescence intensity was quantified (**j**) (*n* = 4). **k**, **l** Anlotinib treatment enhanced intratumoral accumulation of free Dox. 4T1 tumor-bearing mice treated with saline or anlotinib were intravenously administered Dox. The content of Dox in tumor tissue was measured by HPLC 12 h and 24 h post-injection (**k**) (*n* = 4). Fluorescent images of Dox in tumor sections 24 h post-injection are shown (**l**). Scale bar, 100 μm. **m**, **n** SAR131675 treatment enhanced intratumoral accumulation of nanoparticles. 4T1 tumor-bearing mice treated with saline or SAR131675 were intravenously administered Lip-Cy5.5. Tumors were excised 8 h post-administration for ex vivo imaging (**m**), and the average fluorescence intensity was quantified (**n**) (*n* = 3). **o** SAR131675 treatment enhanced intratumoral accumulation of free Dox. 4T1 tumor-bearing mice treated with saline or SAR131675 were intravenously adminisered Dox. The content of Dox in tumor tissue was measured by HPLC 12 h post-injection (*n* = 3). The data are presented as the mean ± s.d. *p < 0.05; **p < 0.01; ***p < 0.001

Previous studies have shown that lymphangiogenesis occurs both at the tumor periphery (peritumoral LVs) and within the tumor mass (intratumoral LVs). It is well acknowledged that LVs are important highways for the metastasis of cancer cells, yet controversy persists regarding the drainage function of intratumoral LVs.^28,38,39^ After observing the phenomenon that intravenously injected nanoparticles (Lip-Rhodamine with a size of ~100 nm, Supplementary Fig. S4) can extravasate from tumor blood vessels (Supplementary Fig. S5), we next investigated whether substances within tumors can be drained through tumor-associated LVs. 4T1 tumor-bearing mice were first intravenously administered Lip-Rhodamine, and 10 μl FITC-dextran was injected into local tumor tissues to label the tumor LVs. Using multiphoton microscopy, we observed that intratumoral FITC-dextran could effectively exit the tumors and proficiently label LVs surrounding the tumors. Meanwhile, we observed that dye-labeled nanoparticles exited the tumors via LVs and ultimately entered the nearby draining LNs (Fig. 3a). Additionally, 0.2% Evans blue was locally injected into the tumor tissue for optical observation. At 2 h post-injection, we observed the evident drainage of the Evans blue into the draining LN through a distinct and well-defined channel (Supplementary Fig. S6). Next, we explored whether anti-lymphangiogenesis could reduce the drainage of substances from the tumor to the draining LN. To this end, we first prepared Cy5.5-labeled liposomes (Lip-Cy5.5) with a size of 100 nm through a thin-film hydration method (Supplementary Fig. S7). Subsequently, we locally injected the liposomes into the tumor tissues of mice that had received saline or anlotinib treatment. Then tumors and draining LNs were surgically isolated 8 h post-injection for fluorescence imaging. The fluorescence signals of the tumor tissues from anlotinib-treated mice were significantly stronger than those from saline-treated group. In contrast, the fluorescence signals of the draining LNs from anlotinib-treated group were significantly weaker than those from saline-treated group (Fig. 3b–d), indicating that the inhibition of lymphangiogenesis contributed to the enhanced intratumoral retention of nanoparticles. In order to visually observe the changes in the distribution of intratumorally injected nanoparticles in LNs after anti- lymphangiogenesis therapy, 100 nm Au nanoparticles (AuNPs) (Supplementary Fig. S8) were intratumorally administered, and the draining LNs were excised at 4 h post-injection and processed into ultrathin sections for transmission electron microscopy (TEM) imaging. Remarkably, the TEM images revealed an obvious reduction in the number of AuNPs that entered draining LNs following treatment with anlotinib and SAR131675 (Fig. 3e, f). These data indicate that substances that accumulate within tumors can be drained by tumor-associated LVs, while anti-lymphangiogenesis therapy contributes to enhanced intratumoral retention.

### Enhanced intratumoral accumulation of nanoparticles, macromolecules, and small molecular drugs after anti-lymphangiogenesis therapy

We subsequently examined whether anti-tumor lymphangiogenesis can enhance the intratumoral accumulation of systemically injected agents. First, commercially available fluorescent nanoparticles with a size of 40 nm, as characterized by TEM and dynamic light scattering (DLS) (Supplementary Fig. S9), were intravenously injected into 4T1 tumor-bearing mice that had been pre-treated with either saline or anlotinib. The intratumoral accumulation of nanoparticles was monitored and quantified using an in vivo imaging system (IVIS) at various time points. The anlotinib-treated group exhibited higher accumulation of nanoparticles in tumors (Fig. 3g–j). Evans blue is reported to specifically bind to albumin in plasma and is often used to assess the tumor accumulation of macromolecule.^3^ Thus, a systemic administration of Evans blue into mice was performed to monitor the intratumoral distribution of albumin. The results showed that anlotinib treatment also augmented the intratumoral accumulation of the Evans blue-albumin complex (Supplementary Fig. S10). Moreover, we intravenously administered the Cy5.5-labeled anti-PD-L1 antibody (PD-L1-Cy5.5), an immune checkpoint inhibitor, into mice pretreated with saline or anlotinib. The fluorescence signal of PD-L1-Cy5.5 within tumors was monitored by IVIS and confocal laser scanning microscopy 4 h post-injection. As indicated in Supplementary Fig. S11, the fluorescence signal of PD-L1-Cy5.5 in tumors was significantly improved in the group treated by anlotinib. We further evaluated the impact of anlotinib treatment on the intratumoral accumulation of small molecular drugs. Mice received saline or anlotinib treatment in advance were intravenously administered with free Dox. At 12 and 24 h post-administration, the concentrations of Dox in tumor homogenates were measured by high performance liquid chromatograph (HPLC) with a fluorescence detector. The data revealed that the concentrations of Dox in the tumor tissues from anlotinib-treated mice were nearly twice as high as those from saline-treated mice (Fig. 3k, l). We also demonstrated the effectiveness of this strategy in non-hyperpermeable Lewis lung carcinoma (LLC) tumor model (Supplementary Fig. S12). SAR131675 was employed to further validate the phenomenon of enhanced intratumoral accumulation of therapeutics following anti-lymphangiogenesis therapy. As shown in Fig. 3m–o, the content of free Dox and Lip-Cy5.5 increased in tumors from mice treated with SAR131675 compared with the saline group. In summary, the anti-lymphangiogenesis therapy mediated by both anlotinib and SAR131675 can significantly increase the tumor accumulation of diverse drug formulations, including nanoparticles, macromolecules, and small molecular drugs.

### Effects of anlotinib and SAR131675 on tumor blood vessels and tumor stroma

**Fig. 4:**
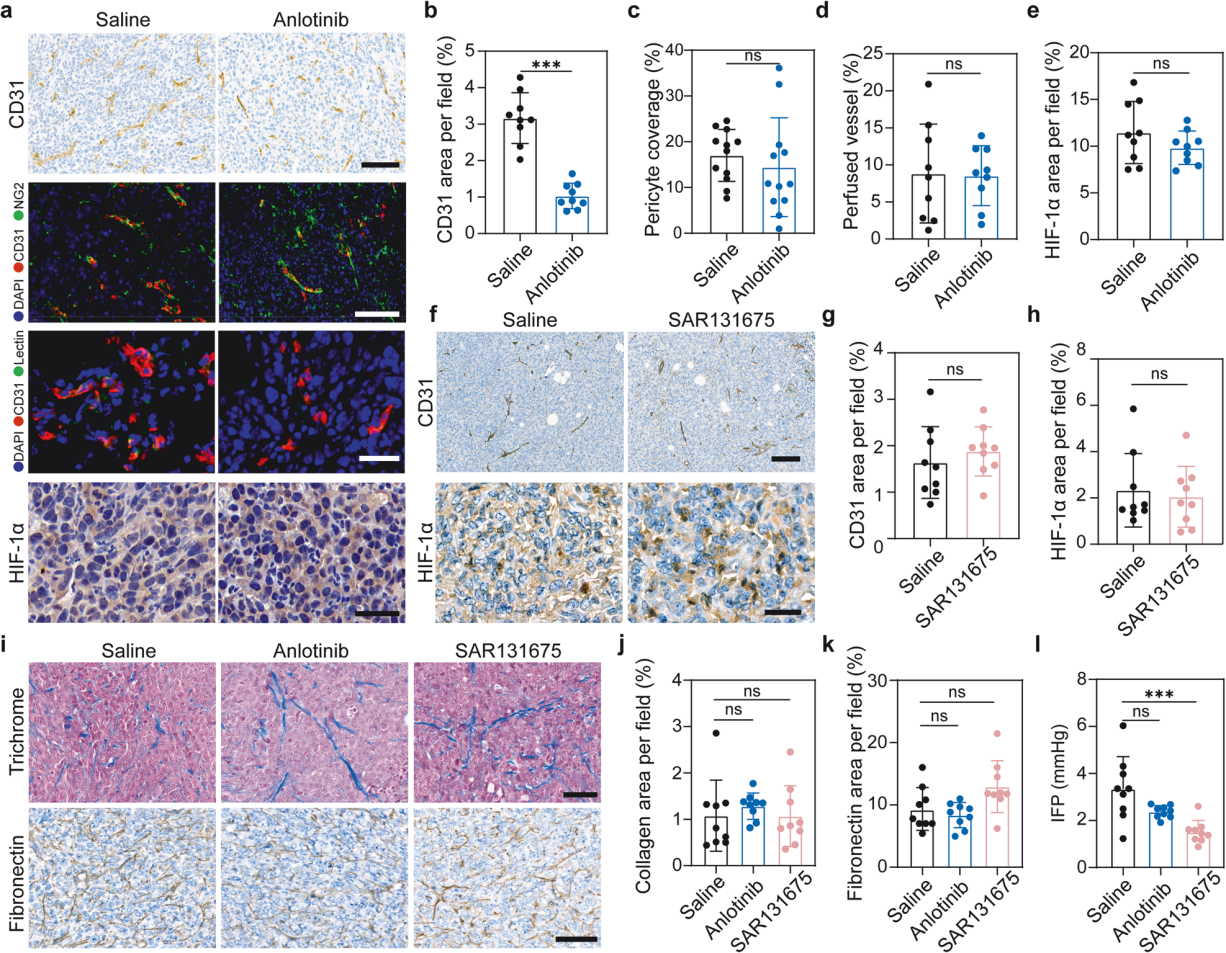
Effects of anlotinib and SAR131675 on the structure and function of tumor blood vessels, tumor stroma and IFP. **a**–**e** During the time and dosing window of anlotinib used in this experiment, tumor vessel normalization did not occur. Immunohistochemistry staining for endothelial cells (CD31, brown) (Scale bar, 100 μm), immunofluorescence staining for endothelial cells (CD31, red) and pericytes (NG2, green) (Scale bar, 80 μm), fluorescence images of Dylight^@^488-lectin-perfused (green) tumor blood vessels (CD31, red) (Scale bar, 40 μm), and representative images of HIF-1α (brown) immunohistochemical staining (Scale bar, 40 μm) in 4T1 tumor sections from mice treated with saline or anlotinib are shown in (**a**). Quantitative analysis of tumor vascular density (**b**), pericyte coverage (**c**; NG2^+^CD31^+^ area percentage of the total CD31^+^ area), perfused vessels (**d**; lectin^+^ CD31^+^ area percentage of the total CD31^+^ area), and HIF-1α area (**e**) as shown in (**a**) (*n* = 9 or *n* = 12; images were from three mice per group). **f**–**h** SAR131675 did not influence the density and function of tumor blood vessels. Immunohistochemistry staining for endothelial cells (CD31, brown) and HIF-1α (brown) in 4T1 tumor sections from mice treated with saline or SAR131675 are shown in (**f**). Scale bar, 100 μm in the upper panels and 20 μm in the lower panels. Quantification of tumor vascular density (**g**) and HIF-1α area (**h**) as shown in (**f**) (*n* = 9; images were from three mice per group). **i**–**k** Anlotinib and SAR131675 did not modulate tumor stroma. Histological studies with trichrome staining of collagen and immunohistochemical staining of fibronectin in tumor (**i**). Scale bar, 50 μm. Quantitative analysis of collagen (**j**) and fibronectin (**k**) (*n* = 9; images were from three mice per group). **l** Tumor IFP of tumor-bearing mice treated with saline, anlotinib, or SAR131675 for 10 consecutive days (*n* = 9). The data are shown as the mean ± s.d. ns no significance, ***p < 0.001

It has been reported that VEGFR-3 also takes part in tumor angiogenesis, and blocking VEGFR-3 signaling with a highly specific VEGFR-3-TKI or monoclonal antibody has shown ability to reduce vascular density.^36,40,41^ In the current work, we employed two VEGFR-3-TKIs to verify our hypothesis. The first TKI, anlotinib, is a clinically approved multi-target TKI with both anti-lymphangiogenesis and anti-angiogenic ability. The second TKI, SAR131675, is a potent and selective VEGFR-3-TKI with anti-lymphangiogenesis ability. When administered with the right dosage and appropriate time window, anti-angiogenic agents have the potential to normalize tumor vasculature, which can enhance the intratumoral delivery of small molecular drugs and nanomedicines in a size-dependent manner.^42,43^ Taking these concerns into account, we next investigated whether treatment with anlotinib or SAR131675 at the employed experimental dosage could induce vasculature normalization. In mice treated with anlotinib, the immunohistochemical staining of tumor sections for the vascular marker CD31 revealed a reduction in the density of tumor blood vessels (Fig. 4a, b). To assess the structural integrity of tumor vasculature, we performed immunofluorescence staining of tumor tissue for both CD31 and pericyte marker, NG2. The acquired images revealed that anlotinib treatment did not increase the pericyte coverage of vasculature in tumor site compared with saline treatment (Fig. 4a, c), indicating that anlotinib did not promote the maturation of the tumor blood vessels. We next evaluated tumor vessel perfusion by intravenous injection of DyLight^@^488-labeled lectin into tumor-bearing mice. Compared with the saline group, anlotinib treatment did not increase the fraction of CD31^+^lectin^+^ vessels (Fig. 4a, d). Another important indicator of tumor blood vessel normalization is the alleviation of hypoxia, so we proceeded to assess HIF-1α expression in tumor tissues via immunohistochemistry staining. Again, no notable difference was observed between the treatment and control group (Fig. 4a, e). In addition, we utilized photoacoustic imaging to assess oxygen saturation (StO_2_) and total hemoglobin (HbT) levels in the tumor tissue. These two factors are the most used indices of oxygenation and blood perfusion, respectively. Our findings revealed that treatment with anlotinib did not enhance the oxygenation or blood perfusion in the tumor tissue (Supplementary Fig. S13). Together, these results suggest that anlotinib does not improve the structure and function of tumor blood vessels, and its effects on tumor vasculature do not contribute to the enhanced intratumoral accumulation of aforementioned substances. In SAR131675-treated mice, there was no significant alteration in tumor vascular density compared to saline-treated mice (Fig. 4f, g), and the relief of tumor hypoxia was also not observed (Fig. 4f, h). In addition, considering the multi-target effect of anlotinib, we conducted a comprehensive assessment of tumor stroma and interstitial fluid pressure (IFP), two critical factors that influence the distribution and penetration of therapeutic drugs in tumor tissue. We first evaluated the levels of collagen and fibronectin, which are two key constituents of tumor stroma, by Masson’s trichrome staining and immunohistochemistry staining, respectively. The results demonstrated that neither anlotinib nor SAR131675 had any impact on tumor stroma (Fig. 4i–k). Moreover, compared with the saline-treated group, there was no significant change in tumor IFP in the anlotinib-treated group, whereas it decreased in SAR131675-treated mice (Fig. 4l, Supplementary Fig. S14). Reports have indicated that the VEGF-C-induced peritumor lymphatics exhibited abnormal function.^28^ One possible reason for the decrease of IFP following SAR131675 treatment is that the anti-lymphangiogenesis activity of SAR131675 normalizes the function of the existing peritumoral LVs which might enhance the drainage of fluid in tumor tissue, leading to a further decrease in intratumoral IFP. With the thought that intratumoral drugs are difficult to penetrate dense tumor stroma, especially for nanoparticles larger than 100 nm in size,^34^ we speculate that the functional restoration of peritumoral LV does not facilitate the exit of drugs from solid tumors. The reduction of IFP after SAR131675 treatment may serve as another factor enhancing intratumoral accumulation of therapeutic drugs. Indeed, further investigation is needed to provide solid evidence and substantiate this hypothesis. Overall, all the results above-mentioned demonstrate that anlotinib treatment reduces vascular density without improving vascular perfusion, and has no effect on tumor stroma or tumor IFP. These findings highlight the distinct mechanisms by which anlotinib and SAR131675 contribute to improved intratumoral drug accumulation. The improved intratumoral accumulation of therapeutic drugs after anlotinib treatment is exclusively reliant on the anti-lymphangiogenesis effect of anlotinib. Although SAR131675 does not affect tumor blood vessels and tumor stroma, it reduces tumor IFP. Consequently, SAR131675 improves the accumulation of therapeutic drugs in tumors through its combined effects of anti-lymphangiogenesis and reduction of tumor IFP.

### Preparation and characterization of Lip-Dox

Inspired by the results obtained above, we speculated that the enhanced intratumoral accumulation resulting from anti-lymphangiogenesis could potentially enhance the anti-cancer efficacy of the nanomedicines, therapeutic antibodies, and small molecular drugs. Therefore, a model nanomedicine of Dox-loaded liposomes (Lip-Dox) was fabricated by the thin-film hydration approach. TEM images show that Lip-Dox possesses a phospholipid bilayer structure with a size of ~100 nm (Supplementary Fig. S15a). DLS analysis indicated that Lip-Dox possessed a hydrodynamic diameter of 93.63 ± 2.7 nm and a negative surface charge of −10.13 ± 0.45 mV (Supplementary Fig. S15b). The Dox release profile of Lip-Dox revealed a 54.4% release of Dox after 42 h of incubation in PBS at physiological pH. However, when the pH was 4.4, mimicking the lysosomal environment, almost 100% of the Dox was released after this duration (Supplementary Fig. S15c). Additionally, the particle size, zeta potential, and morphology changed only negligibly after 11 days at pH 7.4 (Supplementary Fig. S15d, e), suggesting that the prepared Lip-Dox has good stability under physiological pH. To further confirm the cytotoxic efficacy of Lip-Dox, we evaluated the nanoformulation’s cellular uptake and cytotoxicity using free Dox as a control. The cellular uptake of Lip-Dox was confirmed utilizing flow cytometry by incubating free Dox or Lip-Dox of equivalent Dox weight with 4T1 cells for 2 h. As illustrated in Supplementary Fig. S15f, the Dox signals in Lip-Dox- and Dox-treated cells were almost the same. We further observed the cellular uptake using confocal laser scanning microscope. As indicated in Supplementary Fig. S15g, both free Dox and Lip-Dox were taken up by 4T1 cells and co-localized with nuclei. Finally, we investigated the cytotoxicity of Lip-Dox in 4T1 cells by CCK-8 assay. The Lip-Dox samples exhibited similar cytotoxicity to the corresponding concentration of free Dox at the cellular level (Supplementary Fig. S15h).

### Enhanced therapeutic efficacy of Lip-Dox and Dox when combined with anlotinib

**Fig. 5:**
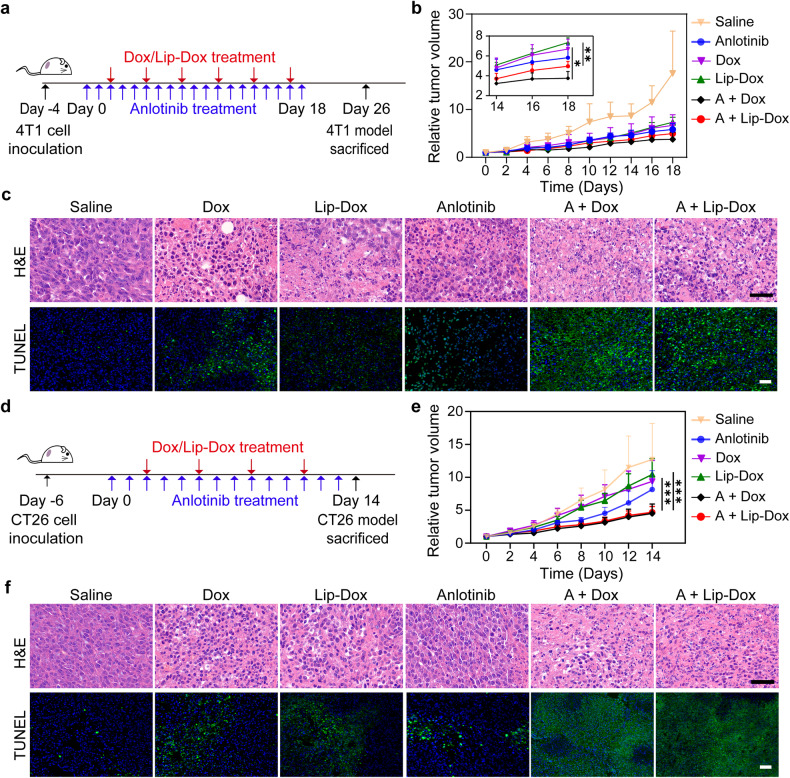
Improved therapeutic efficacies of Dox and Lip-Dox (equivalent Dox dosage: 3 mg/kg) when combined with anlotinib (1.5 mg/kg) in murine 4T1-luc and CT26 xenograft tumor models. **a** Experimental timeline for treatments in the 4T1-luc tumor model. **b** Tumor growth curves of the 4T1-luc tumor model (*n* = 5). **c** Representative images of H&E staining and TUNEL assay of tumor tissues from different treated groups. Scale bar, 40 μm. **d** Experimental timeline for treatments in the CT26 tumor model. **e** Tumor growth curves of the CT26 tumor model (*n* = 7). **f** Histological studies with H&E staining and TUNEL assay in CT26 tumor sections. Scale bars, 40 μm. Data are shown as the mean ± s.d. *p < 0.05; **p < 0.01; ***p < 0.001

With the ability to augment the intratumoral accumulation of therapeutic drugs via anti-lymphangiogenesis, anlotinib holds great potential to enhance the anti-tumor efficacy of multiple drugs. In order to test this hypothesis, we established two subcutaneous murine tumor models based on 4T1-luc and CT26 cells. The tumor-bearing mice were randomly divided into six groups: saline, anlotinib, Dox, Lip-Dox, anlotinib + Dox (A + Dox), and anlotinib + Lip-Dox (A + Lip-Dox). When the tumor volume reached ~100 mm^3^, saline or anlotinib was intraperitoneally administered daily with a total of ten injections. Next, saline, Dox, or Lip-Dox was intravenously administered every three days. The experimental timeline for the treatment of the 4T1-luc tumor model is shown in Fig. 5a. The combination therapeutic groups (A + Dox and A + Lip-Dox) exhibited significantly higher anti-tumor activity than any of the monotherapies (Fig. 5b). Consistent with the tumor growth curves, H&E analysis and TUNEL (deoxynucleotidyl-transferase-mediated nick end labeling) staining showed that tumors from A + Dox- and A + Lip-Dox-treated groups exhibited greater nuclear chromatin condensation and fragmentation, as well as a stronger TUNEL signal, compared with the monotherapy groups (Fig. 5c). For CT26 tumor model, the experimental timeline of which is shown in Fig. 5d, Dox, Lip-Dox, and anlotinib treatment all elicited limited anti-tumor efficacy. Encouragingly, the combination of Dox or Lip-Dox with anlotinib resulted in a greater tumor suppression rate (Fig. 5e), presumably due to the enhanced tumor accumulation of Dox and Lip-Dox after anlotinib treatment. Consistent with the tumor growth curves, treatments with Dox, Lip-Dox, or anlotinib individually did not significantly reduce tumor mass, while anlotinib plus Dox or anlotinib plus Lip-Dox significantly decreased tumor weight in comparison with the corresponding monotherapies (Supplementary Fig. S16). Additionally, H&E analysis and TUNEL staining revealed that the tumor tissues from the combined therapeutic groups showed more apoptosis and necrosis (Fig. 5f), indicating an improved anti-tumor efficacy. During the treatment, none of the treated groups exhibited a significant change in body weight (Supplementary Fig. S17), suggesting that this strategy is relatively safe. We also evaluated the toxicity of the different treatments to organs by H&E staining and serum biochemistry analysis in the CT26 tumor model. As shown in Supplementary Fig. S18, serum biochemistry indicators of heart and liver integrity in the free Dox-containing groups (Dox and A + Dox) markedly changed in comparison with the saline group. When Dox was encapsulated into liposomes (Lip-Dox), such toxicity was significantly reduced. Meanwhile, anlotinib did not aggravate the liver and heart toxicity associated with Dox and Lip-Dox. It is worth noting that when used in combination with anlotinib, the cardiotoxicity of Dox was somewhat reduced. One possible explanation for this is that the enhanced intratumoral accumulation of Dox decreased the amount of Dox in the circulation and organs. H&E staining of the major organs showed no noticeable histological alterations in all experimental groups (Supplementary Fig. S19). Taken together, these data demonstrate that anlotinib treatment enhanced the anti-tumor efficacy of both the nanomedicine and free chemotherapeutic by enhancing drug accumulation in tumor tissue.

### Immunoregulatory effects of anlotinib

**Fig. 6:**
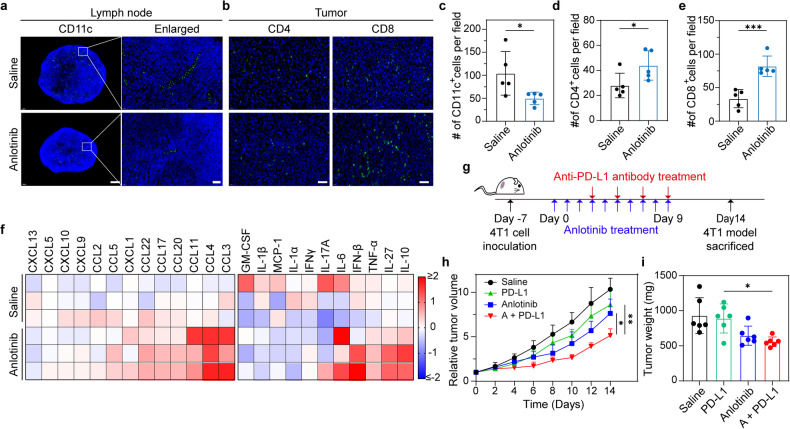
Anlotinib modulates anti-tumor immunity and improves the therapeutic efficacy of anti-PD-L1 checkpoint blockade. **a** Representative immunofluorescence images showing CD11c^+^ DCs in tumor-draining LNs. The nuclei are stained blue, and CD11c is green. Scale bar, 400 µm. Higher magnification is shown to the right of each image. Scale bar, 40 µm. **b** Representative immunofluorescence images of tumor tissues to evaluate CD4^+^ and CD8^+^ T-cell infiltration. The nuclei are stained blue, and CD4 and CD8 are green. Scale bar, 50 µm. **c** Number of CD11c^+^ DCs per field (*n* = 5, images were from three mice per group). **d**, **e** Number of CD4^+^ T cells (**d**) or CD8^+^ T cells (**e**) per field (*n* = 5, images were from three mice per group). **f** Heatmap representing the expression of inflammatory factors and chemokines in tumor tissues from mice treated daily with saline or anlotinib for 10 consecutive days. **g** Experimental timeline for the combined therapy experiment. **h** Tumor growth curves of the 4T1 tumor model (*n* = 6). **i** Tumor weights of excised tumors (*n* = 6). The data are shown as the mean ± s.d. *p < 0.05; **p < 0.01; ***p < 0.001

Tumor-associated LVs play an essential role in anti-cancer immunity. On the one hand, they serve as channels for the transport of tumor antigens to draining LNs, and tumor-associated LECs actively promote dendritic cell (DC) migration toward draining LNs, which are essential for the induction of anti-tumor immunity.^26,44^ On the other hand, inflamed LECs foster an immunosuppressive TME by producing nitric oxide synthase, indoleamine 2,3-dioxygenase, and transforming growth factor-β.^26^ Additionally, it has been widely reported that anti-angiogenic drugs can also enhance the therapeutic efficacy of immunotherapy by modulating TME,^45^ in which the combined use of anti-PD-1/PD-L1 blockade with anti-angiogenic agents has attracted great interest.^46^ Considering the anti-angiogenic and anti-lymphangiogenic activities of anlotinib, we next evaluated the influence of anlotinib on anti-tumor immunity and its effect on the therapeutic efficacy of the anti-PD-L1 antibody in a 4T1 tumor-bearing mouse model. Consistent with expectation, immunofluorescence staining of CD11c in tumor-draining LNs showed that anlotinib treatment reduced DC number in LNs (Fig. 6a, c), which may be attributed to the decreased density of tumor-associated LVs. Nevertheless, the infiltration of CD4^+^ and CD8^+^ T cells within tumors increased after anlotinib therapy **(**Fig. 6b, d, e), consistent with a previous report.^47^ To further evaluate the change in immune response within tumors, we examined the concentrations of various chemokines and cytokines in tumor homogenates. As shown in Fig. 6f, the levels of several chemokines, including the T-cell and NK-cell recruitment-related chemokines, CCL4, CCL20, and CCL3, increased in anlotinib-treated mice. Meanwhile, the concentrations of some pro-inflammatory cytokines, including TNF-α and IL-6, also increased after anlotinib treatment. Taken together, the data indicate that anlotinib treatment can transform tumors into an immune “hot” state.

Combining the impacts of anlotinib on the tumor immune microenvironment and intra-tumor accumulation of the anti-PD-L1 antibody, we next assessed the therapeutic effects of treatment with anlotinib plus anti-PD-L1 antibody in the 4T1 tumor model. The treatment timeline is presented in Fig. 6g. The anti-PD-L1 antibody alone showed limited anti-tumor efficacy, while the combined strategy resulted in significant anti-tumor efficacy (Fig. 6h). There was no noticeable reduction in body weight among any of the treatment groups (Supplementary Fig. S20). At the end of treatment, the weights of the excised tumor tissue were recorded, reflecting the same trends as the tumor growth curves (Fig. 6i).

The migration of antigen-captured DCs toward tumor-draining LNs is critical for T cell activation. In our study, we found that anti-lymphangiogenesis therapy leads to fewer DCs in tumor-draining LNs, potentially impeding anti-tumor T cell responses. However, our findings also showed that this approach enhances the infiltration of CD4^+^ and CD8^+^ T cells in tumor tissue (Fig. 6b). The retained antigen-captured DCs have the potential to directly activate T cells within the TME, contributing to a comprehensive anti-cancer effect. Moreover, PD-L1 expression on DCs is known to be pivotal in modulating T-cell responses via PD-1 interaction within tumor-draining LNs.^48^ This interaction is likely to be weakened by anti-lymphangiogenesis therapy, mainly due to the decreased number of DCs in the tumor-draining LNs. Given that anti-lymphangiogenesis therapy increases intratumoral PD-L1 antibody accumulation and alters the tumor immune microenvironment, its overall impact on the effectiveness of anti-PD-L1 antibodies is complex and warrants further investigation. Collectively, although the anti-lymphangiogenesis efficacy of anlotinib decreases the number of DCs in draining LNs, it exhibits a net immunostimulatory effect, which is evidenced by the increased T cell infiltration and elevated levels of pro-inflammatory cytokines.

### Anti-metastatic effects of anlotinib

**Fig. 7:**
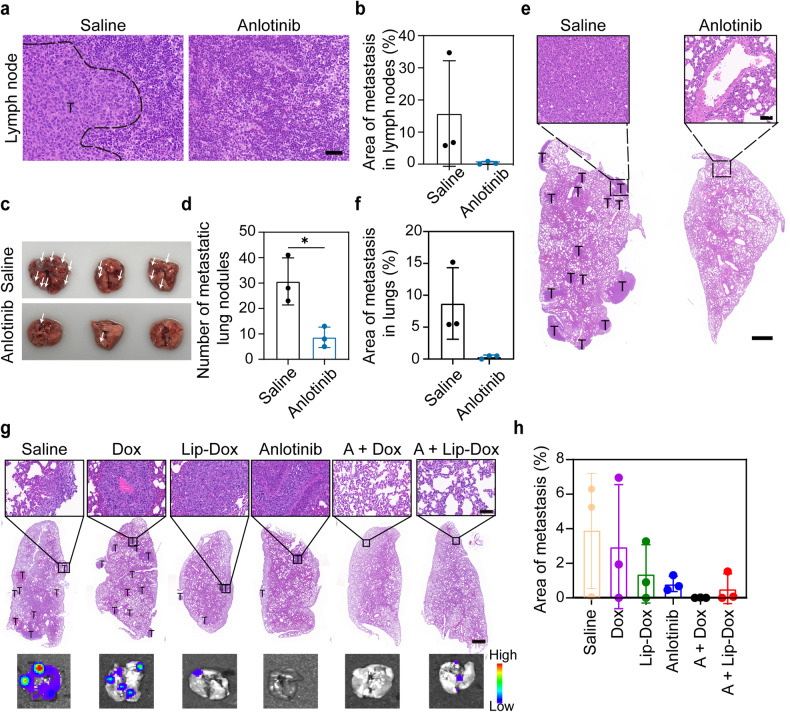
Anti-metastatic efficacy of anlotinib in the murine 4T1 tumor model. Mice were treated daily with saline or anlotinib for 10 consecutive days from day 7 after tumor cell inoculation. The mice were sacrificed 32 days after tumor cell inoculation and tumor-draining LNs and lungs were isolated for further examination. **a** Representative images of H&E-stained tumor-draining LN sections from the indicated treatment groups. Scale bar, 40 μm. **b** Quantification of the area of metastases in the tumor-draining LNs (*n* = 3). **c** Image of excised lungs showing metastases (white arrowheads). **d** Quantification of the number of metastatic nodules in each lung (*n* = 3). **e** Analysis of lung metastases by H&E staining. Scale bar, 1 mm. The upper panels show enlarged images of the indicated areas of interest. Scale bar, 50 μm. **f** Quantification of the area of metastases in lung sections (*n* = 3). **g**, **h** The anti-metastatic activity of combined therapy in the 4T1-luc tumor model. H&E-stained lung tissue sections and the representative bioluminescence images of metastatic foci (**g**). Scale bar, 1 mm. The upper panels show enlarged images of the indicated areas of interest. Scale bar, 80 μm. Quantification of the areas of metastases in the indicated treatment groups (*n* = 3) (**h**). The data are shown as the mean ± s.d. *p < 0.05

In several types of cancer, lymphangiogenesis within tumor tissue is closely linked to the metastasis and is often indicative of poor outcomes.^29,30,49,50^ Clinical data have shown that the occurrence rate of tumor metastasis through LVs is three to five times greater than that through blood vessels, and anti-lymphangiogenesis therapy has been identified as a promising approach to decreasing the incidence of tumor metastasis.^23,38,51–53^ In this study, we further investigated the anti-metastatic activity of anlotinib in a murine 4T1 breast tumor model. Tumor-bearing mice were administered daily with saline or anlotinib for 10 consecutive days. Mice were sacrificed once the tumor volume of the saline group reached 2000 mm^3^. The inguinal LNs and lungs were harvested. As shown in Fig. 7a, b, a large area of metastatic lesions was present in the draining LNs of the saline group, while there was no apparent tumor colonization in the draining LNs of the anlotinib group. Since lung tissue is the preferential organ of metastasis for 4T1 tumors, we collected the lungs from the mice for further histological analysis. The images of the lungs in Fig. 7c, d show that there were a reduced number of metastatic lesions in the anlotinib group than in the saline group. H&E staining also corroborated these results (Fig. 7e, f).

In addition, we observed lung metastasis in the combinatorial therapy experiment in the 4T1-luc tumor model. Eight days after the final treatment, the mice received an intraperitoneal injection of D-luciferin potassium salt. The lungs were collected, and the metastatic foci were visualized by examining the bioluminescence of 4T1-luc tumor cells using an IVIS. We also performed H&E staining of the lung tissues to further examine metastatic foci. As shown in Fig. 7g, h, the treatment groups containing anlotinib exhibited significant inhibition of metastasis, as indicated by the decreased bioluminescence intensity and metastasis area. Collectively, these results demonstrate that anlotinib blocked tumor colonization in draining LNs and prevented distal metastasis.

## Discussion

The inadequate accumulation of therapeutic agents in tumor tissue leads to unsatisfactory therapeutic efficacy and is also one of the most important obstacles to overcome prior to the clinical translation of nanomedicines. To improve drug penetration in tumor tissue, recent endeavors have been directed toward modulating tumor blood vessels or stroma. However, these strategies may introduce new concerns, such as the likelihood of promoting tumor metastasis. Developing multi-functional integrated nano-carriers to improve drug permeability in tumor tissue represents another promising strategy. However, their complex structures bring new challenges for large-scale production. Thus, there remains an urgent need to explore more tractable clinical methods to enhance drug accumulation at the tumor site. In the current work, we proposed a strategy to enhance drug accumulation within tumor tissue by regulating tumor-associated LVs, which also effectively inhibit tumor metastasis.

With tumor development, lymphangiogenesis and LV remodeling occur, leading to the formation of new LVs both within and surrounding the tumor tissue, further facilitating tumor progression. Increasing evidence has indicated that tumor-associated LVs in tumor and peripheral tissues play vital roles in the transport of substances from tumors to draining LNs. In particular, the drainage of nanoparticles from tumor tissue via tumor-associated LVs has been demonstrated. Besides, these LVs also serve as an important pathway for the dissemination of tumor cells, rendering them attractive therapeutic targets.

In the current study, we explored the influence of anti-tumor lymphangiogenesis on drug accumulation in tumor tissue. Our findings indicated that drugs within tumor tissue can be drained by tumor-associated LVs. Anlotinib, a multi-target TKI with anti-lymphangiogenesis activity, and SAR131675, a selective VEGFR-3 TKI with potent anti-lymphangiogenesis activity can significantly inhibit the proliferation, migration and tubule formation of hLECs in vitro, and decrease the density of LVs in vivo. Treatment with the two drugs improves the intratumoral accumulation of various drug formulations, including nanoparticles, macromolecules, and small molecular drugs. Additionally, the in vivo synergism experiments indicate that anti-lymphangiogenesis can significantly improve the anti-tumor efficacy of both nanomedicine and free molecular drugs. Meanwhile, according to the hematologic examination results, the organ toxicity of free molecular drugs in the combined treatment group was significantly decreased, which can be attributed to the enhanced drug intratumoral accumulation and decreased organ distribution. It is noteworthy that such strategy not only improves the tumor accumulation of nanomedicines but also enhances the accumulation of macromolecular drugs and small molecular drugs, surpassing the size limitation of vasculature normalization to nanoparticles. We also excluded the potential effects of anlotinib on tumor blood vessels, tumor stroma, and IFP, and concluded that the improved drug accumulation was not caused by blood vessel normalization or stroma depletion but rather a reduction in tumor-associated LVs. Additionally, SAR131675 showed no effect on tumor blood vessels and tumor stroma, but it led to a reduction of tumor IFP, which could be attributed to the function normalization of the existing peritumoral LVs. This effect can improve fluid drainage from tumor tissues, contributing to the decrease of IFP. However, intratumoral drugs are often trapped within tumor tissue by the dense stroma, making it a challenge for their transport to the peritumoral LVs. Consequently, this mechanism is unlikely to significantly facilitate the drainage of drugs from tumors through LVs. Furthermore, the reduction of IFP has great potential to enhance the deep penetration of intratumoral drugs. In summary, SAR131675 has capability to enhance intratumoral drug accumulation through anti-lymphangiogenesis and reduction of tumor IFP. Additionally, the anti-tumor efficacy of the anti-PD-L1 antibody was significantly improved by anlotinib through the enhanced accumulation of antibodies in tumor tissue and modulation of anti-tumor immunity. Our results also demonstrated that anti-lymphangiogenesis can effectively inhibit lymphatic system-associated tumor metastasis either by monotherapy or combined therapy.

Although we have demonstrated the effectiveness of anti-lymphangiogenesis in enhancing intratumoral drug accumulation, there remain several aspects worthy of further exploration. In the current work, we observed impaired perfusion of tumor blood vessels after anlotinib treatment, which is a known limitation on drug penetration and accumulation in tumors.^41^ Considering the fact that the density of LVs in tumor site is not as high as blood vessels, we propose that an anti-lymphangiogenesis strategy is suitable for combination with tumor blood vessel regulation strategies to boost maximum drug accumulation. The reduced tumor IFP following SAR131675 treatment highlights the possibility that the inhibition of lymphangiogenesis could lead to alterations in lymphatic function. This phenomenon deserves further exploration. In addition, identifying the optimal size range of nanoparticles which is suitable for this strategy is essential and deserves additional studies. Literature has reported LVs also play important roles in shaping tumor immune-microenvironment. On the one hand, LVs serve as channels for the transport of tumor antigen to draining LNs, and the LECs promote DC migration toward draining LNs by secreting chemokines, which is critical for the generation of anti-tumor immune response. On the other hand, the inflamed LECs in TME also produce immunosuppressive molecules, fostering an immunosuppressive TME. In our study, we observed that anti-lymphangiogenesis treatment resulted in a reduction in the number of DCs in tumor-draining LNs. However, the broader implications of this strategy on anti-tumor immune response within the LNs remain unclear, and whether it impacts the effectiveness of DC vaccines in eliciting a robust immune response remains to be verified. Therefore, evaluating the effects of anti-lymphangiogenesis on anti-tumor immune response in detail is of great significance. Even though we have explored the immunoregulatory effects of anlotinib in the current work, it should be noted that its effects cannot fully represent those of anti-lymphangiogenesis therapy owing to the multi-target feature of anlotinib. Therefore, more specific inhibitors of lymphangiogenesis, such as SAR131675, are needed to investigate their effects on immune regulation and further understand the distinct contributions of anti-lymphangiogenesis to tumor immunology.

In summary, we describe a promising strategy to improve the accumulation of therapeutic drugs in tumor site. Anti-tumor lymphangiogenesis not only improves the anti-tumor efficacy of therapeutic drugs but also effectively suppresses tumor metastasis, therefore showing great potential for combination with other mainstream therapeutic modalities.

## Materials and methods

### Materials and reagents

Anlotinib dihydrochloride was a gift from Jiangsu Chia-Tai Tianqing Pharmaceutical Co., Ltd. SAR131675 was purchased from TargetMol (USA). PBS, RPMI 1640 medium, penicillin-streptomycin, and fetal bovine serum (FBS) were purchased from Wisent (Canada). The BCA protein assay kit and 40 nm FluoSpheres^TM^ Carboxylate-Modified Microspheres were obtained from Thermo Fisher Scientific (USA). HSPC, cholesterol, and DSPE-PEG2000 were purchased from Lipoid GmbH (Germany), J&K (China), and AVT (China), respectively. RIPA lysis buffer, phenylmethanesulfonyl fluoride (PMSF), Evans blue, and DAPI were obtained from Solarbio Life Science (China). Cy5.5 was purchased from Meryer (China). Rhodamine 6 G was obtained from Aladdin (China). FITC-dextran was purchased from Sigma-Aldrich (USA). Cell counting kit-8 (CCK-8) was purchased from Dojindo Molecular Technologies (Japan). Doxorubicin hydrochloride was purchased from MESO (China). Recombinant human VEGF-C (C546) was obtained from Novoprotein (China). Antibodies against LYVE-1 (ab14917), HIF-1α (ab16066), NG2 (ab129051), fibronectin (ab2413) were obtained from Abcam (UK). An antibody against LYVE-1 (103-PA50) was purchased from ReliaTech (Germany). Antibodies against VEGFR-3 (AF4201) and p-VEGFR-3 (AF3676) were purchased from Affinity Biosciences (USA). Antibodies against Akt (4691 T), p-Akt (4060 T), Erk (4695 T), p-Erk (4377 T) were obtained from cell signaling technology (USA). Antibodies against CD31 (GB123151), CD11c (GB11059), CD4 (GB11064) and CD8 (GB114196), and fluorescein (FITC) TUNEL cell apoptosis detection kits were purchased from Servicebio (China). Dylight^@^488-labeled lycopersicon esculentum (Tomato) lectin (100 μl in PBS) was purchased from Vector Laboratories (USA). Anti-PD-L1 antibody was obtained from BioXcell (USA). A Mouse Proinflammatory Chemokine Panel (13-plex) with a V-bottomed plate (740451) and a Mouse Inflammation Panel (13-plex) with a V-bottomed plate (740446) were purchased from BioLegend (USA).

### Cell culture

The murine breast cancer cell line 4T1 and the murine colon cancer cell line CT26 were obtained from the American Type Culture Collection. The human lymphatic endothelial cell line (hLECs) was purchased from iCell, China. The luciferase-expressing 4T1 cell line (4T1-luc) was generated by our group using a lentivirus transfection system from Addgene. Mycoplasma contamination was routinely tested negative. CT26 and 4T1 cell lines were cultured in RPMI 1640 supplemented with 10% FBS, penicillin (100 units ml^−1^), and streptomycin (100 μg ml^−1^) at 37 °C in a humidified incubator with 5% CO_2_. hLECs was cultured in primary endothelial cell culture medium purchased from ScienCell (USA).

### Animals

BALB/c mice (female, 6–8 weeks old, 16–18 g body weight) were purchased from Vital River Animal Laboratories Biotechnology Co., Ltd. (Beijing, China) and housed under specific sterile pathogen-free conditions. All animal protocols were approved by the Animal Care and Welfare Committee of the National Center for Nanoscience and Technology. The 4T1 and CT26 xenograft tumor models were established by subcutaneously injecting 4T1 or CT26 cells (1 × 10^6^ cells) into the right flank of mice. The injected cells were suspended in a 100 μl mixture of PBS and Matrigel (1:1; BD, USA). Tumor size was measured every 2 days with a digital caliper. The volume of tumor was determined by the following formula: V = L × W^2^/2, in which L represents the maximum tumor diameter, and W represents the minimum tumor diameter. When the tumors reached ~100 mm,^3^ the mice underwent treatment with different drug formulations. Tumor-bearing mice received anlotinib treatment at a dose of 1.5 mg/kg daily via intraperitoneal injection. For SAR131675 treatment, tumor-bearing mice were given 100 mg/kg SAR131675 orally every day.

### Interference of VEGF-C-mediated activation of VEGFR-3 and downstream molecules in hLECs

To assess the impact of anlotinib and SAR131675 on VEGFR-3 phosphorylation, hLECs were pretreated with the two drugs at different concentrations (0, 0.1, 1 μg/ml), followed by stimulation with VEGF-C (200 ng/ml). The whole protein of hLECs was extracted using RIPA buffer containing 1.0 mM PMSF within 15 min. The whole protein concentration was quantified utilizing a BCA protein assay kit. Cell lysates were first separated by SDS-PAGE and then transferred onto PVDF membranes for further analysis. The membranes were initially blocked in 5% milk or BSA, and then incubated with primary antibody at 4 °C overnight. Subsequently, the membrane was washed and incubated with an HRP-conjugated secondary antibody (Santa Cruz Biotechnology, USA) for 1 h at room temperature. Detection of bands was achieved using the enhanced chemiluminescence reagents. GAPDH was used as an internal control.

### Cell viability assay

hLECs or 4T1 cells were seeded at a density of 5 × 10^3^ cells per well in 96-well cell culture plates and incubated for 24 h. To evaluate the effects of anlotinib and SAR131675 on the proliferation of hLECs, the cells were exposed to various concentrations of anlotinib, SAR131675, or PBS. For the cytotoxicity assays with Lip-Dox, 4T1 cells were incubated with various concentrations of Lip-Dox or the corresponding concentration of Dox. After 24 h incubation, the CCK-8 assay kit was utilized to determine cell viability.

### Wound healing assay

hLECs were placed in 12-well cell culture plates at a density of 1.5 × 10^5^ cells per well until fully confluent. Scratch wounds were made perpendicular to the cell layer with a sterile 200 μl pipet tip. The fixed observation position in each well was marked on the bottom of the dish with a marker. After being washed by PBS for three times, the cells were then incubated with various concentrations of anlotinib or SAR131675 for 15 h. The wounds were imaged by bright-field microscopy (Leica, Germany). The recovered area was measured at three repeated wells using ImageJ software.

### Tubule formation assay

hLECs were seeded into 48-well plates pre-coated with 100 μL Matrigel per well and exposed to various concentrations of anlotinib or SAR131675 for 20 h. For each experimental condition, three parallel groups were set up. Tubule formation was imaged under an inverted light microscope. Tube length was quantified using ImageJ software.

### In vivo anti-tumor lymphangiogenesis assay

When the size of tumors in 4T1 tumor-bearing mice reached approximately 100 mm,^3^ mice were treated daily with saline, anlotinib or SAR131675 for 10 consecutive days. Then tumors were dissected and immersed in 4% paraformaldehyde (PFA) for fixation, and immunohistochemical staining for LYVE-1 (103-PA50) was performed to show the distribution of LVs in tumors. Sections were observed using an inverted light microscope, and the LVs density was analyzed by ImageJ software. In addition, 4T1 or CT26 tumors from mice treated with saline and anlotinib for 10 consecutive days were dissected, embedded in OCT, and stored at −80 °C for subsequent immunofluorescence assays. Immunofluorescence staining of LYVE-1 (ab14917) was employed to analyze the density of LVs. All sections were photographed with a fluorescence microscope and quantitatively analyzed utilizing ImageJ software with the same threshold settings.

The expression levels of LYVE-1 were detected through Western blot analysis. Tumor tissues from mice treated daily with anlotinib, SAR131675 or saline for 10 consecutive days were surgically excised and mechanically disrupted in RIPA buffer with 1.0 mM PMSF. The following steps are the same with the above-mentioned method. Anti-LYVE-1 antibody (103-PA50) was used as the primary antibody. GAPDH was chosen as an internal control.

### Evaluation of lymphatic drainage

To assess the drainage function of tumor-associated LVs, 4T1 tumor-bearing mice, 15 days after inoculation, were intravenously injected with 100 μl Lip-Rhodamine solution. 4 h later, the mice were anesthetized and intratumorally administered with 10 μl FITC-dextran 2,000,000 (25 mg/ml) to label tumor-associated LVs. Then, the inguinal LN adjacent to the tumor was surgically exposed, and the drainage of Lip-Rhodamine from the tumor to draining LN was observed by multiphoton laser scanning microscopy (Olympus, Japan). For the observation of the drainage of intratumoral material from the tumor to draining LN, 30 μl 0.2% Evans blue was locally injected into tumor tissue. 2 h later, the mice were euthanized, and the drainage of Evans blue from tumor tissue into the draining LN was imaged under an inverted light microscope.

### Preparation and characterization of Au nanoparticles (AuNPs)

AuNPs in size of 100 nm were prepared based on the previously described protocols.^54^ Specifically, 600 μl fresh NaBH_4_ solution (10 × 10^−3^ M) was injected into 10 ml deionized water with HAuCl_4_ (0.25 × 10^−3^ M) and cetyltrimethylammonium bromide (CTAB, 100 × 10^−3^ M) under vigorous stirring. After incubation at 27 °C for 3 h, 50 μl mixture was mixed with 2 ml cetyltrimethylammonium chloride (CTAC, 200 × 10^−3^ M) and 1.5 ml ascorbic acid (AA, 100 × 10^−3^ M) followed by one-shot injection of 2 ml HAuCl_4_ solution (0. 5 × 10^−3^ M), obtaining 10 nm AuNPs. The nanoparticles were obtained by centrifugation at 14,500 rpm for 25 min, followed by a single rinse with deionized water, and then stored in 1 ml CTAC solution (20 × 10^−3^ M). 10 μl 10 nm seed solution was mixed with 2 ml CTAC (100 × 10^−3^ M) and 130 μl AA (10 × 10^−3^ M), then 2 ml HAuCl_4_ (0.5 × 10^−3^ M) was dropwise added into the solution at a speed of 2 mg h^−1^. The reaction was kept for 10 min under 27 °C, obtaining 46 nm AuNPs. The product was collected by centrifugation at 14,500 rpm for 10 min. After being washed by deionized water, 46 nm AuNPs were suspended in 0.86 ml of deionized water. Lastly, 0.5 ml 46 nm seed solution was mixed 2 ml CTAC (100 × 10^−3^ M) and 130 μl AA (10 × 10^−3^ M), and 2 ml HAuCl_4_ (0.5 × 10^−3^ M) was dropwise added into the solution at a speed of 2 mg h^−1^. The reaction was kept for 10 min under 27 °C. After centrifugation at 14,500 rpm for 10 min, 100 nm AuNPs were collected. Washed the product once with deionized and stored it in 0.86 ml deionized water.

The morphology of 100 nm AuNPs was observed by TEM (HT7700, HITACHI, Japan) at an 80 kV acceleration voltage. To prepare the samples for TEM, 10 μl of diluted AuNPs were deposited onto a carbon-coated copper grid. The surface plasmon absorption of AuNPs was detected by UV-Vis absorbance spectrophotometry (PerkinElmer, USA). The hydrated size, polydispersity index (PDI), and zeta potential of 100 nm AuNPs were determined utilizing a Malvern Zetasizer Nano ZS90 (Malvern, UK). Three measurements for each sample were performed.

### Evaluation of the effects of anti-tumor lymphangiogenesis on lymphatic drainage of intratumoral nanoparticles

4T1 tumor-bearing mice were treated with anlotinib or saline for 10 consecutive days. 30 μl Cy5.5-labeled liposomes were locally injected into tumor tissues. 8 h later, tumors and the inguinal LNs adjacent to the tumors were dissected, and the fluorescence intensities of the nanoparticles in the LNs and tumors were measured using an IVIS. For TEM observation, mice bearing 4T1 tumors treated with anlotinib, SAR131675 or saline for 10 consecutive days were intratumorally injected with 100 nm Au nanoparticles. At 4 h post-injection, the inguinal LNs adjacent to the tumors were dissected and sectioned into fragments approximately 1 mm.^3^ After fixation with 2.5% glutaraldehyde in H_2_O (pH 7.2–7.4), rinse with buffer, and post-fixation with 1% OsO_4_ in PB (0.1 M, pH 7.4), the pieces were dehydrated through a graded ethanol series gradient. Then pieces were encased in resin and subjected to polymerization. 60–80 nm slices were prepared using ultramicrotome and stained with uranyl acetate and lead citrate. The cuprum grids were observed using TEM.

### Evaluation of the influence of anlotinib/SAR131675 treatment on drug accumulation in tumor tissue

Mice bearing 4T1 tumors were treated daily with anlotinib or saline for 10 consecutive days and then intravenously injected with 100 μl of diluted 40 nm Fluospheres^TM^ Carboxylate-Modified Microspheres (1:133), Cy5.5-labeled anti-PD-L1 antibody (0.5 mg for each mouse), or 0.2% Evans blue (10 mg/kg). Then the mice were imaged through an IVIS at various time intervals, and fluorescence intensities of the tumor tissues were quantified. In order to evaluate the effect of anlotinib treatment on Dox accumulation in tumor tissue, 4T1 tumor-bearing mice received saline or anlotinib pre-treatment were injected with Dox (8 mg/kg) through the tail vein. At 12 h and 24 h post-injection, tumors were surgically isolated. Pieces of tumors (~200 mg) were mechanically disrupted in 500 μl RIPA lysis buffer. Next, 500 μl acetonitrile was added into the lysate, followed by incubation overnight at 4 °C. The mixture was then centrifuged at 14,000 × *g* at 4 °C for 30 min. The supernatant was obtained, and the concentration of Dox was determined by HPLC with a fluorescence detector at an excitation wavelength of 480 nm and emission wavelength of 590 nm. Samples obtained from untreated tumors were used as blank controls. For evaluation of the influence of SAR131675 treatment on intratumoral accumulation of small molecular drugs and nanoparticles, 4T1 tumor-bearing mice pretreated with saline or SAR131675 were injected with Dox (8 mg/kg) or Lip-Cy5.5 through the tail vein. For mice administered with Dox, Dox concentration in each tumor was measured utilizing HPLC 12 h post-injection. For mice administered with Lip-Cy5.5, the tumors were excised 8 h post-injection and imaged using an IVIS.

### Evaluation of tumor blood vessels and tumor stroma

To evaluate the influence of anlotinib on the structure and function of tumor blood vessels, tumor tissues from mice treated daily with saline or anlotinib for 10 consecutive days were collected for further analysis. The density of tumor blood vessels was evaluated by immunohistochemistry (IHC) staining for the endothelial cell-specific marker CD31 (GB123151). Anti-NG2 (ab129051) and anti-CD31 (GB123151) antibodies were used to analyze the pericyte coverage of tumor vasculature by immunofluorescence staining. The percentage of pericyte-covered blood vessels was defined as the percentage of NG2^+^CD31^+^ area to the total CD31^+^ area. Immunofluorescence staining of cryosections was performed to investigate the perfused tumor blood vessels. Mice were injected with Dylight^@^488-labeled lectin through the tail vein. 10 min post-injection, tumors were harvested and cut into 15 µm sections after being embedded in OCT. An anti-CD31 (GB123151) antibody was used to label tumor blood vessels, and vessel perfusion was observed through a Zeiss LSM 710 confocal microscope. The proportion of perfused vessels was defined as the percentage of lectin^+^CD31^+^ area to the CD31^+^ area. Tumor hypoxia was analyzed by IHC staining using an anti-HIF-1α antibody (ab16066). To evaluate the impact of SAR131675 on tumor vasculature, tumors were excised from mice treated daily with saline or SAR131675 for 10 consecutive days. The density of tumor blood vessels and tissue hypoxia were evaluated using IHC staining for the CD31 (ab182981) and HIF-1α (ab16066), respectively. To assess changes in tumor stroma after anlotinib/SAR131675 treatment, Masson’s trichrome staining for collagen and IHC staining for fibronectin (ab2413) were performed on paraffin sections of tumors from mice treated with saline, anlotinib, or SAR131675 for consecutive 10 days. The results were analyzed using ImageJ software.

### Photoacoustic imaging

Mice bearing 4T1 tumors were pretreated with saline or anlotinib for 10 consecutive days. Following anesthesia with isoflurane, the mice were placed on a heat pad. Then, transparent and bubble-free ultrasound gel was applied to the tumor area. The ultrasound and photoacoustic imaging system (Vevo LAZR-X, Japan) was employed for image acquisition and quantification. Photoacoustic signals were acquired under excitation at wavelengths of 750 nm and 850 nm, respectively.

### Evaluation of the tumor interstitial fluid pressure (IFP)

Mice bearing 4T1 tumors were pretreated for 10 consecutive days with saline, anlotinib, or SAR131675. A saline-filled tube connected a needle to the pressure measuring system. After intraperitoneal anesthesia, the needle was carefully inserted into the center of tumor, and this step would cause a transient fluctuation in pressure. When the pressure stabilized, the data in the computer were recorded. The pressure signals within the tumors were converted into electric signals via a pressure transducer (PowerLab, ADInstruments) and recorded by LabChart 8 software.

### Synthesis of Lip-Dox and fluorescent dyes-labeled liposomes

Lip-Dox was fabricated by the lipid thin-film hydration method. HSPC, Chol, and DSPE-PEG2000 were dissolved in ethanol at a molar ratio of 11.3:7.7:1. The ethanol was then evaporated under vacuum, resulting in a transparent film at the bottom of the flask. The dried lipid film was rehydrated with 2 ml PBS containing 2 mg Dox, followed by sonication to form a clear suspension. Then the suspension was extruded through a liposome extruder with 200 nm, 100 nm, and 50 nm pore size filters. The excess free Dox was removed by centrifugation at 5000 × *g* for 30 min through a 10 kDa molecular weight cut-off ultrafiltration device (Millipore, USA). For the preparation of fluorescent dyes-labeled liposomes, Rhodamine, lipophilic Cy5.5 was dissolved in ethyl alcohol together with HSPC, Chol, DSPE-PEG2000. Then ethyl alcohol was evaporated under vacuum, and the dried lipid film was rehydrated with 2 ml PBS, followed by successive extrusion through a liposome extruder with 200 nm, 100 nm, and 50 nm pore size filters. The liposome solutions were concentrated by centrifugation at 5000 × *g* for 30 min through a 10 kDa molecular weight cut-off ultrafiltration device (Millipore, USA).

### Characterization of Lip-Dox and fluorescent dyes-labeled liposomes

The morphologies of Lip-Dox and fluorescent-dyes-labeled liposomes were characterized by TEM operating at an 80 kV acceleration voltage. To prepare the TEM samples, 10 μl of diluted suspended nanoparticles (1:100) was deposited onto a carbon-coated copper grid and subjected to negative staining with 1% uranyl acetate. Hydrated size, PDI, and zeta potential of Lip-Dox and fluorescent dyes-labeled liposomes were determined by dynamic light scattering utilizing a Malvern Zetasizer Nano ZS90 (Malvern, UK). Three measurements for each sample were performed.

For the determination of the drug encapsulation and loading efficiency of Lip-Dox, an equivalent volume of DMSO was mixed with freshly prepared Lip-Dox suspension followed by sonication. The supernatant containing Dox was collected after centrifugation at 18,516 × *g* for 10 min, and the content of Dox encapsulated in Lip-Dox was calculated by measuring the absorbance of the supernatant at 485 nm utilizing a UV–vis spectrophotometer (Lambda650, PerkinElmer, USA). To determine the drug loading efficiency, Lip-Dox suspension was freeze-dried, and the total mass of the nanoparticles was measured. The following formulas were employed to determine the encapsulation efficiency (EE) and loading efficiency (LE): EE = (weight of loaded drug) / (weight of initially added drug) × 100%; LE = (weight of loaded drug in the nanoparticle)/(weight of the nanoparticle) × 100%.

To evaluate Dox release profile of Lip-Dox, 1 ml freshly prepared Lip-Dox suspension was placed in a dialysis bag (molecular weight cutoff: 3.5 kDa) and dialyzed against 30 mL PBS (pH 4.4 or 7.4). This process was conducted at 37 °C with the system being orbital shaken at 200 rpm. At specific time intervals, 1 ml of the dialysis buffer was taken out for the purpose of Dox quantification, and concurrently, another 1 mL PBS adjusted to the corresponding pH was added to the system. The amount of released Dox was determined by measuring the absorbance at 485 nm via UV–vis spectrometry.

Flow cytometry and confocal microscopy were utilized for assessment of the cellular uptake of Lip-Dox. For flow cytometry experiments, 4T1 tumor cells were plated in 6-well plates with a seeding density of 5 × 10^3^ cells per well and incubated overnight. The cultured cells were exposed to Lip-Dox or the corresponding concentration of Dox for 2 h. The cells were then collected for flow cytometry analysis using a BD Accuri C6 (BD, USA). Gating strategies are provided in Supplementary Fig. S21. For confocal microscopy, 4T1 tumor cells were placed in 8-well confocal microscopy dishes at a seeding density of 5 × 10^3^ cells per well. After 24 h of incubation, Lip-Dox or the corresponding concentration of Dox was added, and samples were incubated for another 2 h. The cells were fixed by 4% paraformaldehyde for 15 min at room temperature, and the nuclei were stained with DAPI prior to imaging with a Zeiss LSM 710 confocal microscope.

### In vivo combinatorial therapy of anlotinib with Dox or Lip-Dox

The anti-tumor efficacy of anlotinib in combination with nanomedicine or free drug was investigated in 4T1-luc and CT26 subcutaneous xenograft tumor models. Mice bearing 4T1-luc tumors with a size of ~100 mm^3^ were divided at random into six groups (*n* = 5), and received anlotinib or saline daily by intraperitoneal injection for 19 consecutive days. The mice were also treated with Dox or Lip-Dox (the administered Dox dosage was equivalent to 3 mg/kg) or saline through intravenous injection every 3 days. The tumor volume and body weight were monitored every other day. On day 26, three animals were euthanized and treated with D-luciferin potassium salt by intraperitoneal injection. At 10 min post-injection, the lungs of these mice were surgically isolated to observe the metastatic foci via an IVIS. Additionally, the excised lungs and tumors were preserved in 4% paraformaldehyde. Next, the tissues were encased in paraffin and sectioned into 5 μm slices for H&E and TUNEL staining. For the CT26 xenograft tumor model, the tumor tissues were surgically isolated on day 14 (*n* = 7), weighed, and fixed for H&E and TUNEL staining.

### Anti-metastatic effects of anlotinib

Mice bearing 4T1 tumors with a size of ~100 mm^3^ were treated by anlotinib or saline by daily intraperitoneal injection for 10 consecutive days. Once the tumors reached 2000 mm^3^ in volume, the experiment ended, and the mice were sacrificed. Tumor-draining LNs as well as lungs were isolated for H&E staining and imaging.

### Hematologic examination

Whole blood was harvested from CT26 tumor-bearing mice treated with different drug regimens through retro-orbital bleeding. The serum was separated for blood biochemistry analysis through centrifugation at 3000 rpm for 15 min. Liver function was evaluated via the measurement of alanine aminotransferase (ALT) and aspartate transaminase (AST) levels. Heart function was evaluated via measuring plasma lactate dehydrogenase (LDH), lactate dehydrogenase isoenzyme 1 (LDH1), creatine kinase (CK), and creatinine kinase isoenzyme MB (CK-MB).

### Evaluation of anti-tumor immunity

Tumors and tumor-draining LNs of 4T1 tumor-bearing mice treated daily with saline or anlotinib for 10 consecutive days were excised for further analysis. IHC staining for CD11c was performed to evaluate the number of DCs in tumor-draining LN paraffin sections. IHC staining for CD4 or CD8 was performed to evaluate CD4^+^ and CD8^+^ T cell infiltration in tumors. The results were analyzed by ImageJ software.

In order to evaluate the expression levels of proinflammatory chemokines and inflammatory cytokines, tumors from mice treated daily with saline or anlotinib for 10 consecutive days were harvested and homogenized in PBS. Next, the tumor homogenates were analyzed using pro-inflammatory chemokine and inflammatory cytokine detection kits, according to the instructions.

For investigation of the effects of anlotinib on the therapeutic efficacy of the anti-PD-L1 antibody, mice bearing 4T1 tumors of approximately 100 mm^3^ were divided into four groups randomly (*n* = 6) and administered daily with anlotinib or saline by intraperitoneal injection for 10 consecutive days. At the same time, the mice received anti-PD-L1 antibody (0.75 mg/kg) or saline administration through intravenous injection every 2 days starting on day 3. The tumor volumes and body weights were monitored every 2 days. On day 14, the mice were euthanized, and the tumor tissues were surgically isolated and weighed.

### Statistical analysis

The data were analyzed using GraphPad Prism 8. The data are presented as the mean ± standard deviation (mean ± s.d.). Significant differences between two groups were identified using unpaired student’s *t*-test. Comparisons among multiple groups employed one-way analysis of variance (ANOVA) with Tukey’s post-hoc test. Statistical significance was acknowledged when the two-sided *p* < 0.05.

### Supplementary information

Supporting Information

## Data Availability

The essential data supporting the major conclusions of this study are present in the paper or the Supplementary Material. Request for additional information related to the study can be directed to the authors.
