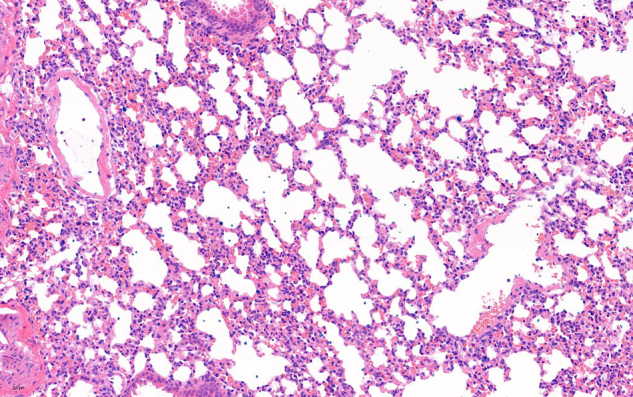# Correction: Anti-lymphangiogenesis for boosting drug accumulation in tumors

**DOI:** 10.1038/s41392-024-01883-4

**Published:** 2024-06-08

**Authors:** Chunling Wang, Junchao Xu, Xiaoyu Cheng, Ge Sun, Fenfen Li, Guangjun Nie, Yinlong Zhang

**Affiliations:** 1 CAS Key Laboratory for Biomedical Effects of Nanomaterials & Nanosafety, CAS Center for Excellence in Nanoscience, National Center for Nanoscience and Technology, Beijing, 100190 China; 2 Sino-Danish College, University of Chinese Academy of Sciences, Beijing, 100190 China; 3 Sino-Danish Center for Education and Research, Beijing, 100190 China; 4 Department of Bioengineering, University of Pennsylvania, Philadelphia, PA 19104 USA; 5 School of Nanoscience and Engineering, University of Chinese Academy of Sciences, Beijing, 100049 China

**Keywords:** Cancer microenvironment, Cancer therapy

Correction to: *Signal Transduction and Targeted Therapy* 10.1038/s41392-024-01794-4, published online 15 April 2024

After the article was published online,^1^ the authors noticed one inadvertent mistake in Supplementary Fig. S19. The image of lung H&E in the Lip-Dox group was repeatedly inserted as the image of Dox group by mistake during the preparation of the figures. The correct figure was provided as follows. The correction did not affect any of our results or discussion presented in the original publication. We apologize for this inadvertent mistake.

The original article has been corrected.
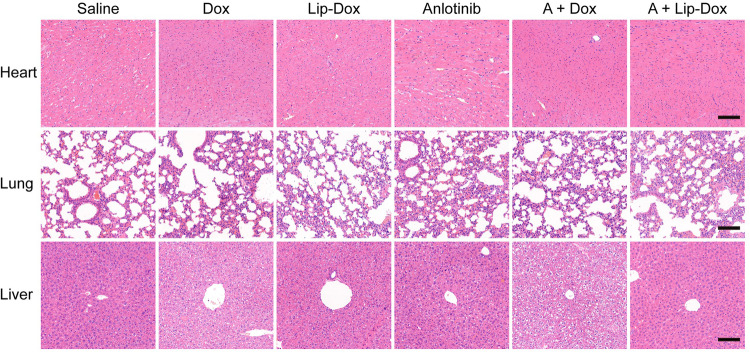


Original uncropped data

H&E staining of lung tissue for Dox group
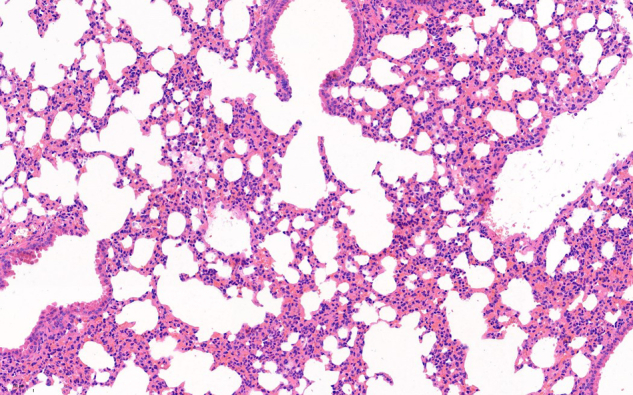


H&E staining of lung tissue for Lip-Dox group