# Supplementary material for: Anti-lymphangiogenesis for boosting drug accumulation in tumors
Source: Signal Transduct Target Ther. 2024 Apr 15;9:89. doi: 10.1038/s41392-024-01794-4 (PMC11016544; doi:10.1038/s41392-024-01794-4)

Supplementary Materials for

Anti-lymphangiogenesis for boosting drug accumulation in tumors

Chunling Wang^1,2,3^, Junchao Xu^1,4^, Xiaoyu Cheng^2,5^, Ge Sun^2,5^, Fenfen Li^1^, Guangjun Nie^1,2,3*^, Yinlong Zhang^2,5*^

Correspondence to: [niegj@nanoctr.cn](mailto:niegj@nanoctr.cn) or zhangyinlong@ucas.ac.cn

**This PDF file includes:**

Figures. S1 to S22


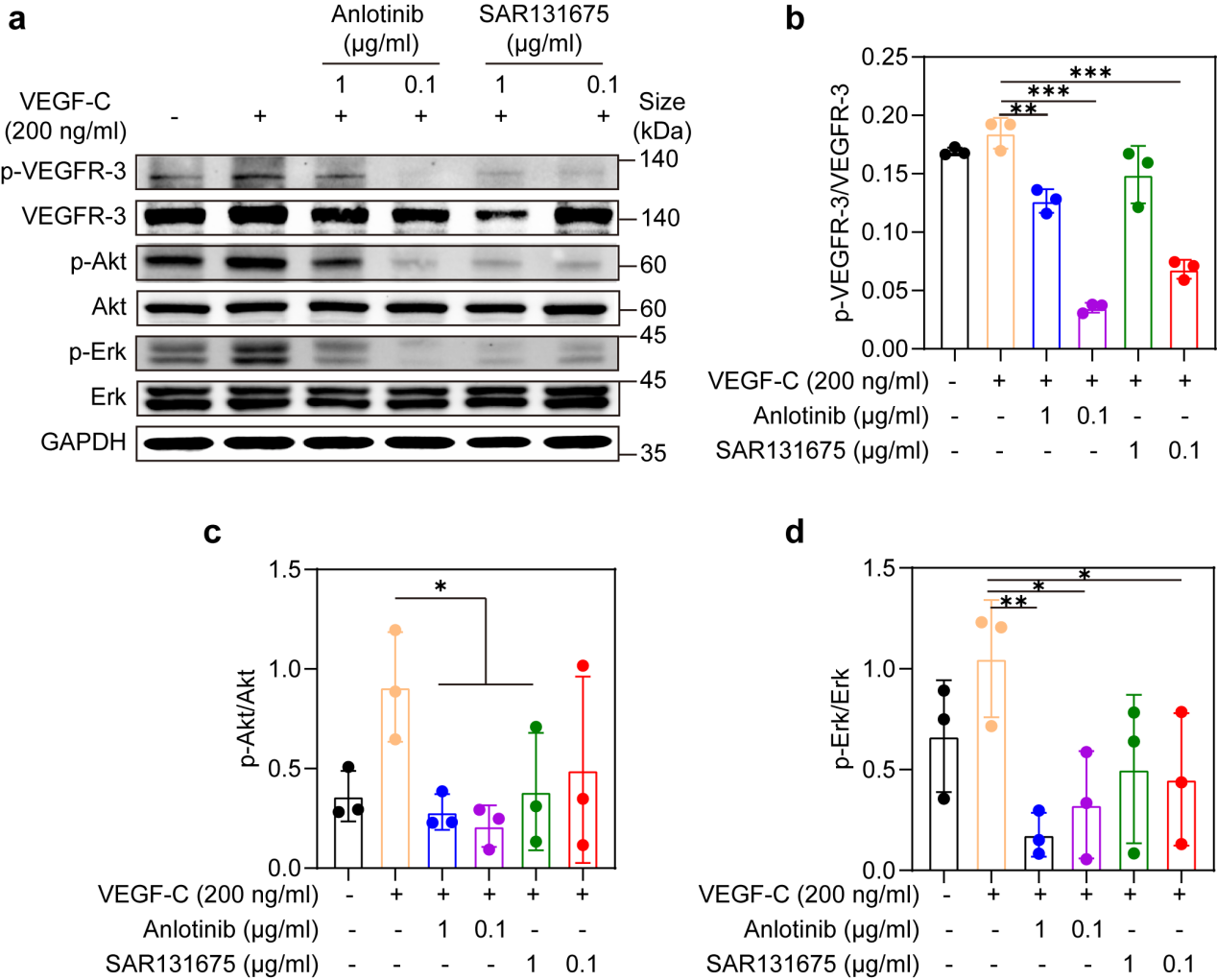


Fig. S1 The blocking of VEGF-C-mediated activation of VEGFR-3 signal pathway. **(a)** Immunoblot analysis of VEGFR-3, Erk, Akt and their phosphorylation in hLECs activated by VEGF-C (200 ng/ml) after treatment with anlotinib or SAR131675. **(b-d)** Densitometry analysis of VEGFR-3 phosphorylation and VEGFR-3 (b), Akt phosphorylation and Akt (c), Erk phosphorylation and Erk (d) bands in (a). The data are shown as the mean ± s.d. (n *=* 3). **p* < 0.05; ***p* < 0.01; ****p* < 0.001.


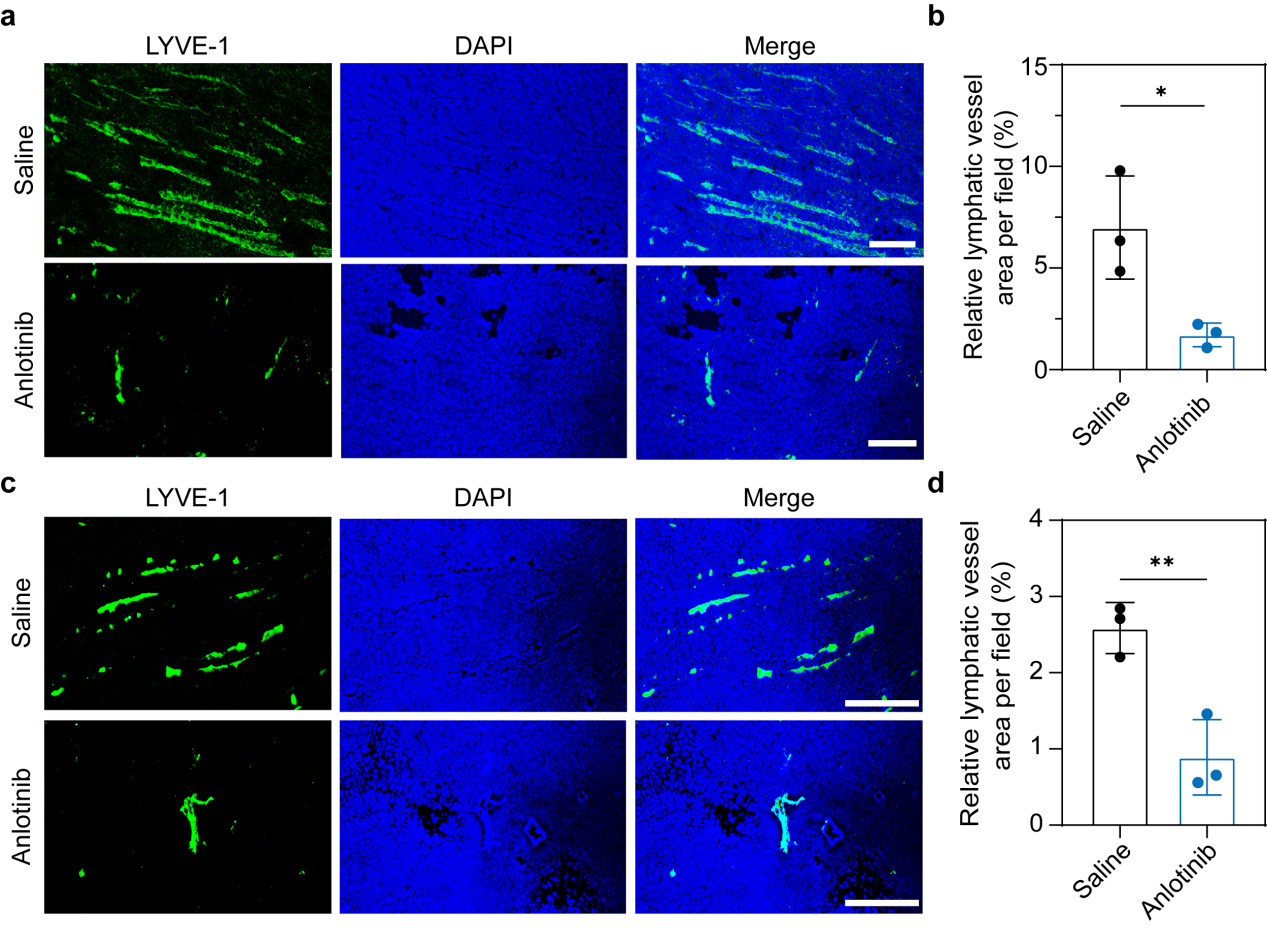


Fig. S2 Anti-lymphangiogenesis activity of anlotinib *in vivo* **(a)** Immunofluorescence staining of 4T1 tumor tissue from mice treated daily with saline or anlotinib for 10 consecutive days. Lymphatic vessels were stained with LYVE-1 (green), and nuclei were stained with DAPI (blue). Scale bar, 100 µm. **(b)** Quantification of the LYVE-1 signal area as shown in figure a using ImageJ (n = 3). **(c)** Immunofluorescence staining of CT26 tumor tissues from mice treated daily with saline or anlotinib for 10 consecutive days. Scale bar, 100 µm. **(d)** LYVE-1 signal area as shown in in figure c was quantified using ImageJ (n = 3). The data are presented as the mean ± s.d. **p* < 0.05, ***p* < 0.01.


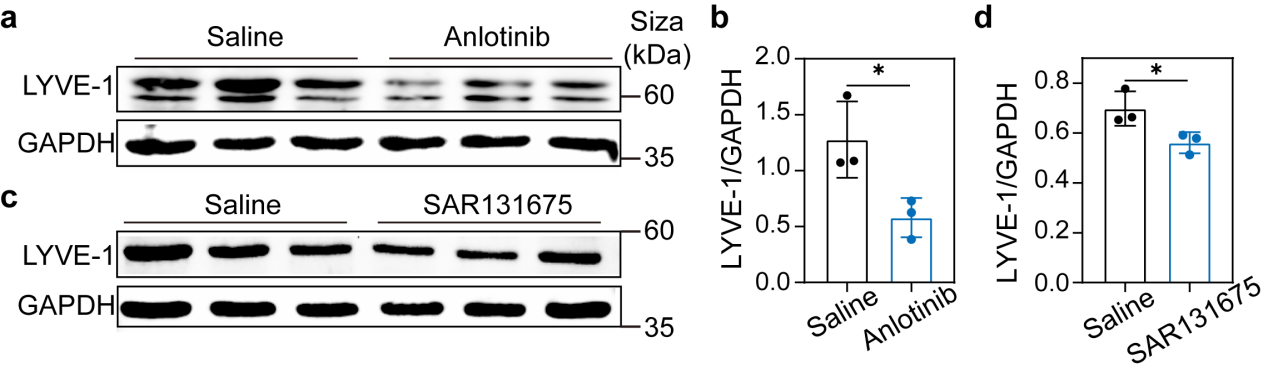


Fig. S3 Western blot analysis of the expression of LYVE-1 in 4T1 tumor tissues from mice treated with saline, anlotinib or SAR131675. Mice were treated daily for 10 consecutive days. GAPDH was used as an internal control (n = 3). **p* < 0.05.


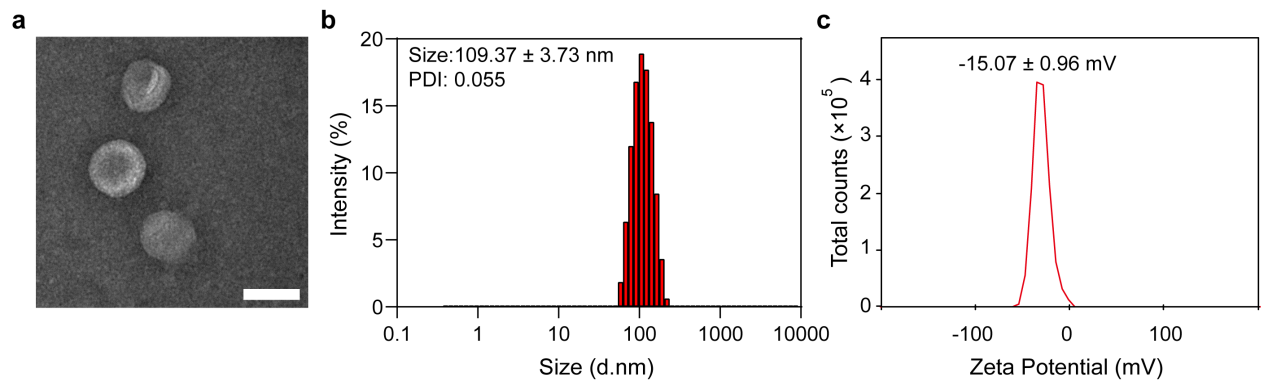


Fig. S4 Characterization of Lip-Rhodamine. **(a)** TEM image of Lip-Rhodamine. Scale bar, 100 nm. **(b-c)** Size distribution (b) and Zeta-potential (c) of Lip-Rhodamine, as determined by dynamic light scattering (DLS; n = 3).


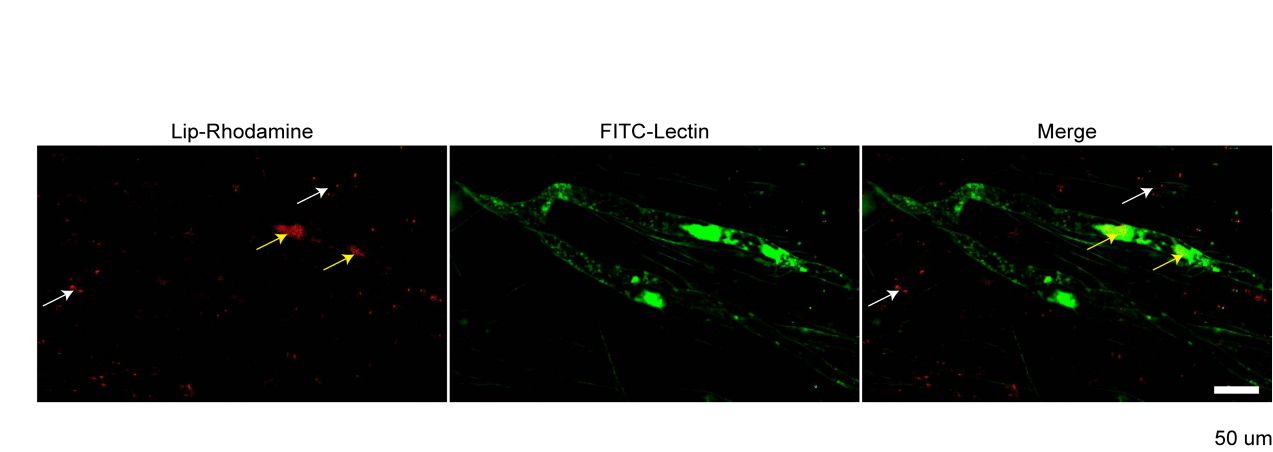


Fig. S5 Representative multiphoton laser scanning microscopy images of tumor blood vessels and the escape of Lip-Rhodamine. Lip-Rhodamine was intravenously administrated into tumor-bearing mice. At 8 h post-injection, mice received an intravenous dose of 100 μl DyLight@488-lectin (1 mg/ml) to label tumor blood vessels. Yellow arrows mark the location of nanoparticles inside the tumor blood vessels, while white arrows point to nanoparticles located outside of these vessels. Scale bar, 50 μm.


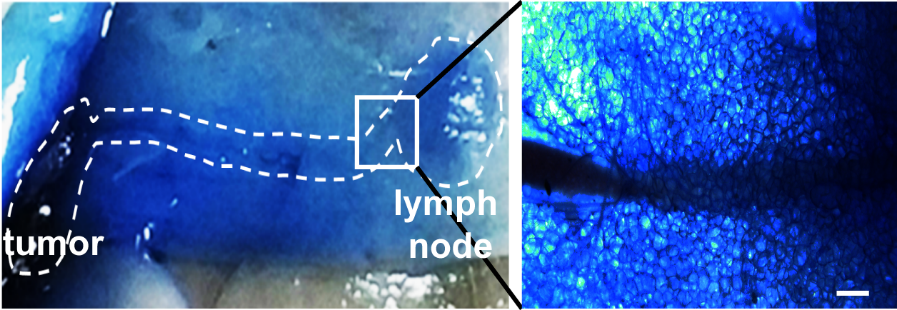


Fig. S6 Representative images of the drainage of tumor-associated lymphatic vessels for Evans blue. Scale bar, 200 μm.


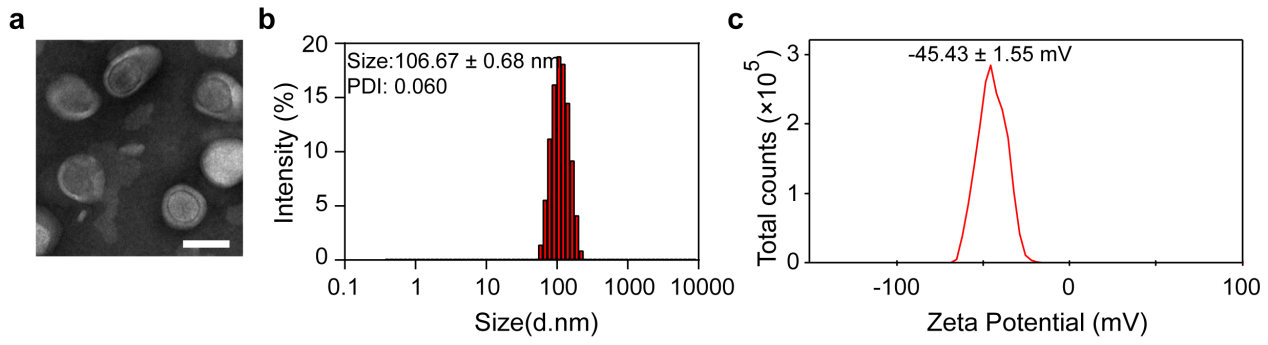


Fig. S7 Characterization of Lip-Cy5.5. **(a)** TEM image of Lip-Cy5.5. Scale bar, 100 nm. **(b-c)** Size distribution (b) and Zeta-potential (c) of Lip-Cy5.5, as determined by DLS (n = 3).


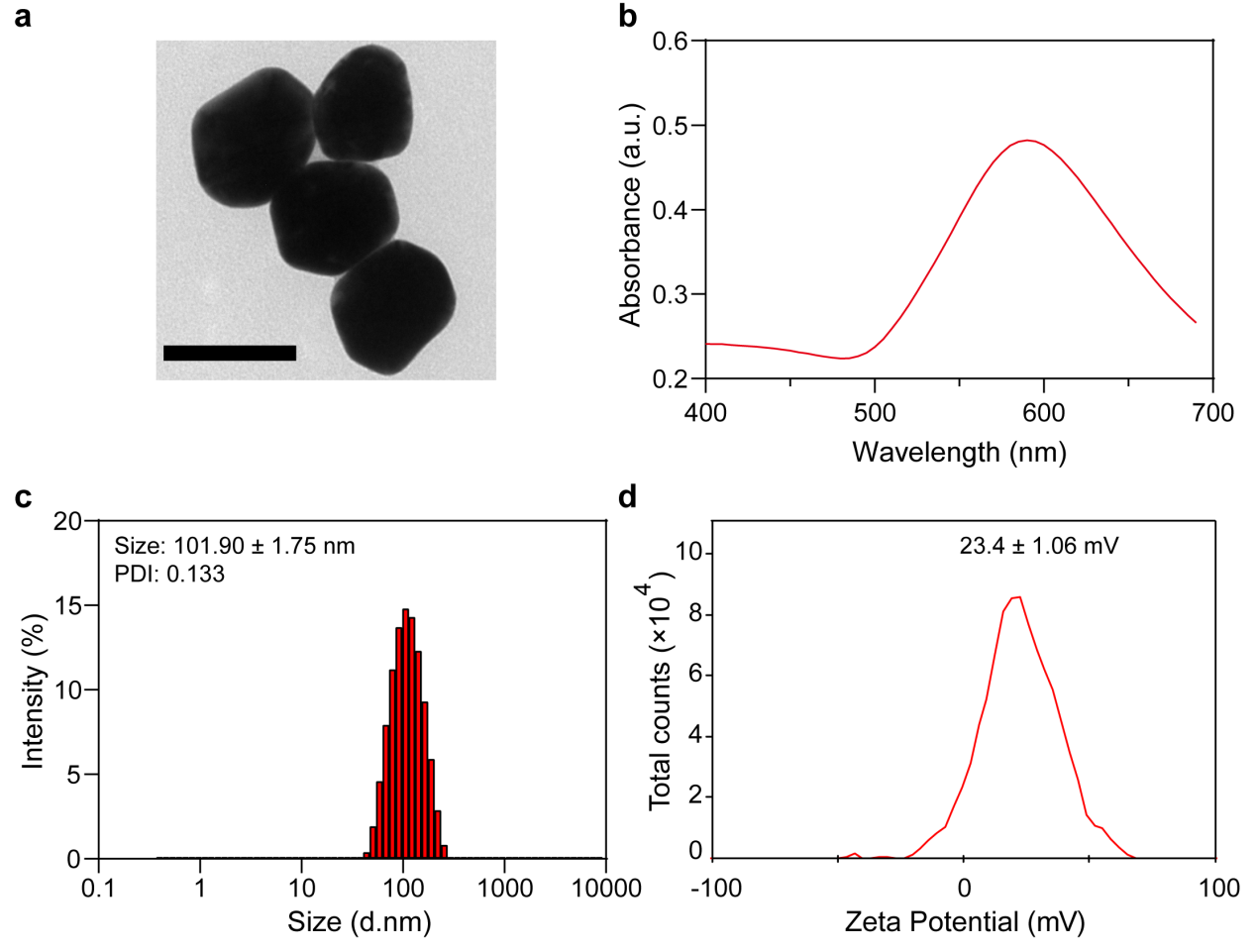


Fig. S8 Characterization of 100 nm AuNPs. **(a)** TEM image of AuNPs. Scale bar, 100 nm. **(b)** Surface plasmon absorption of AuNPs detected by UV-Vis absorbance spectrophotometry. **(c-d)** Size distribution (c) and Zeta-potential (d) of AuNPs, determined by DLS (n = 3).


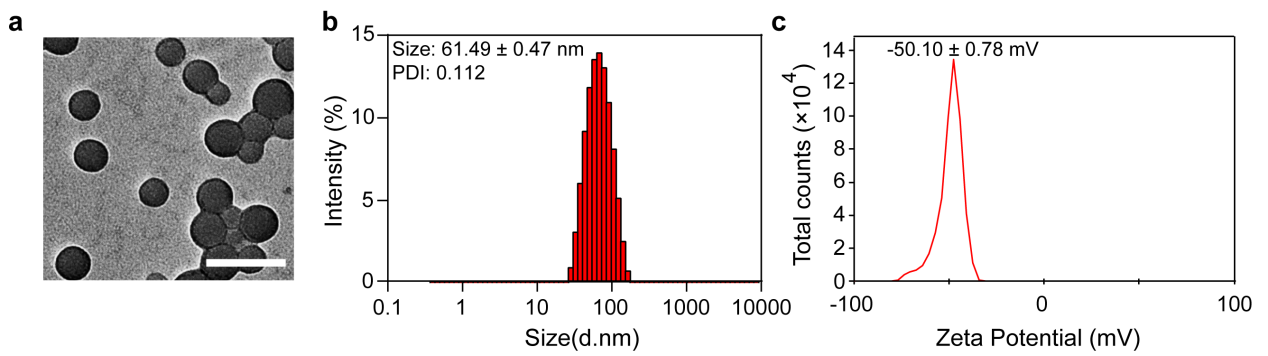


Fig. S9 Characterization of Fluospheres Carboxylate-Modified Microspheres **(a)** TEM image of microspheres. Scale bar, 100 nm. **(b-c)** Size distribution (b) and Zeta-potential (c) of microspheres, as determined by DLS (n = 3).


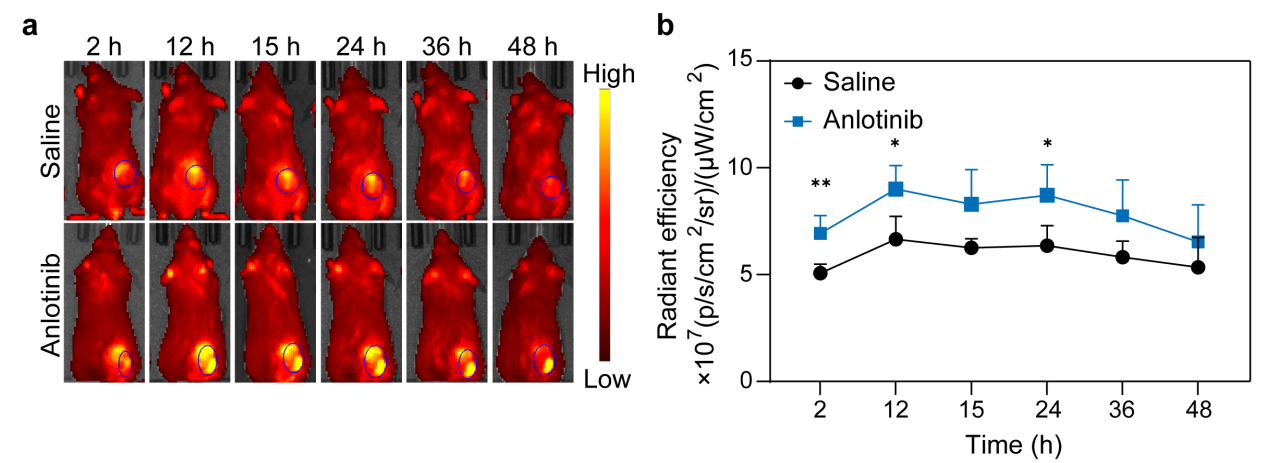


Fig. S10 Accumulation of Evans blue-albumin complexes at the tumor site. **(a)** Representative *in vivo* fluorescence images at the indicated time points. Tumors are circled in blue. **(b)** Quantification of the tumor fluorescence at the indicated time points (n = 4). The data are shown as the mean ± s.d. **p* < 0.05; ***p* < 0.01.


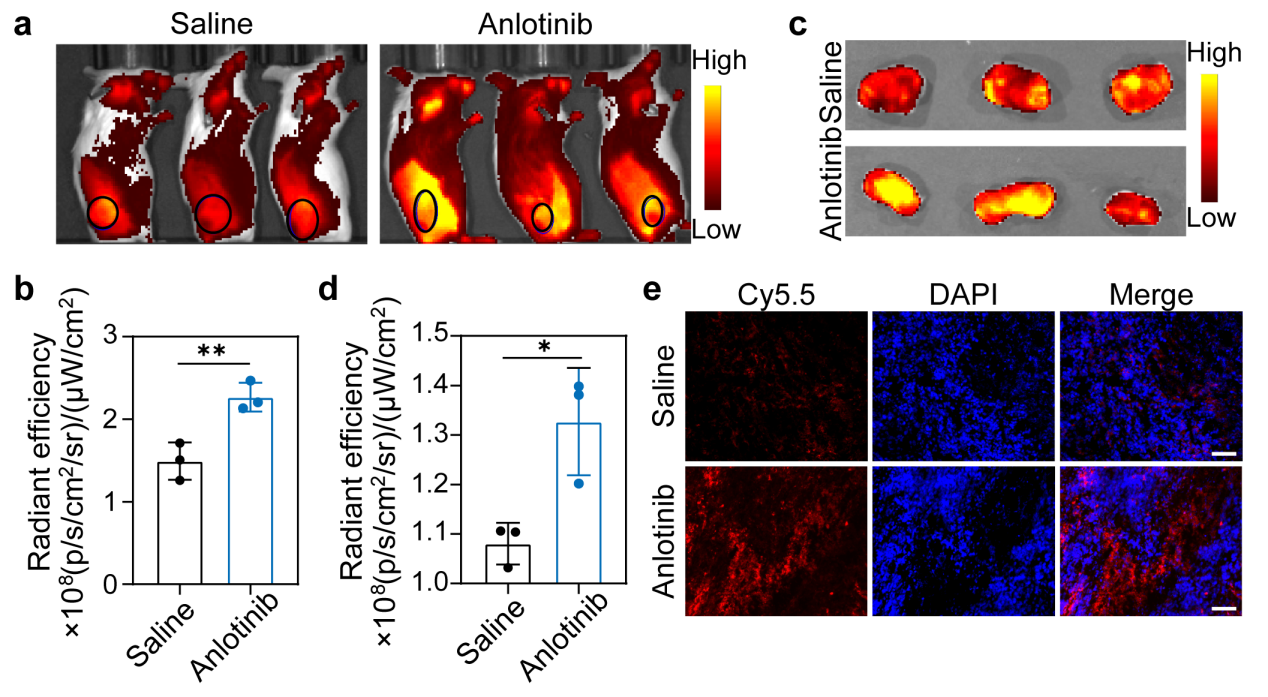


Fig. S11 Accumulation of anti-PD-L1 antibody at the tumor site. **(a-b)** Mice bearing 4T1 tumors were pretreated daily with saline or anlotinib for 10 consecutive days. *In vivo* fluorescence images 4 h post-injection of Cy5.5-labeled anti-PD-L1 antibody are shown, with the tumors circled in black (a). The average tumor fluorescence intensities were quantified (n = 3) (b). **(c-d)** Tumors were excised 4 h post-injection for *ex vivo* imaging (c), and the average fluorescence intensity was quantified (n = 3) (d). **(e)** Distribution of Cy5.5-labeled anti-PD-L1 antibody (red) in tumors at 4 h post-injection. Nuclei were stained with DAPI (blue). Scale bar, 100 μm. The data are shown as the mean ± s.d. **p* < 0.05; ***p* < 0.01.


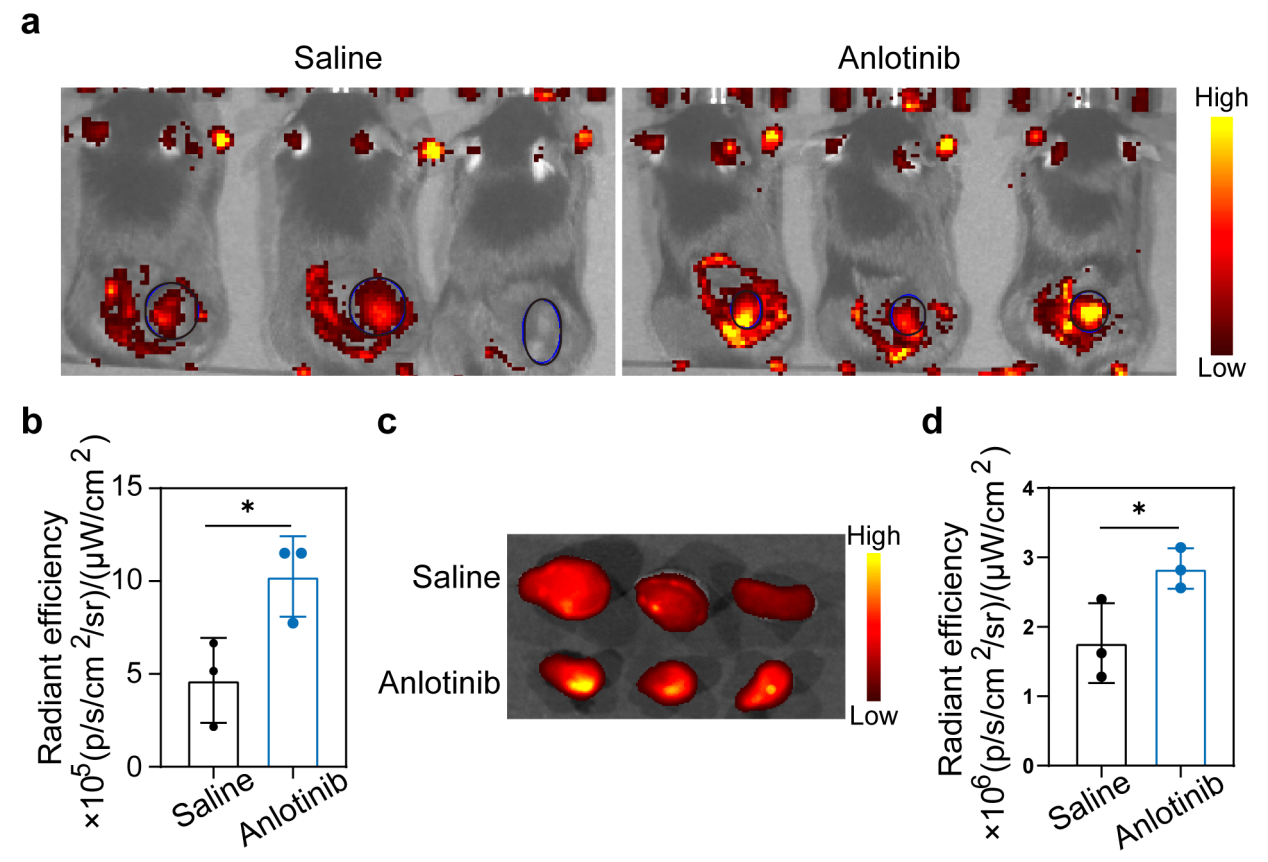


Fig. S12 Accumulation of Lip-Cy5.5 at the tumor site. **(a-b)** Mice bearing LLC tumors received daily pretreatments of either saline or anlotinib for 10 consecutive days. *In vivo* fluorescence images were captured at 8 hours after the injection of Lip-Cy5.5. The tumor regions were outlined in black (a). The average tumor fluorescence intensities were quantified (n = 3) (b). **(c-d)** Tumors were excised 8 h post-injection for *ex vivo* imaging (c), and the average fluorescence intensity was quantified (n = 3) (d). The data are shown as the mean ± s.d. **p* < 0.05.


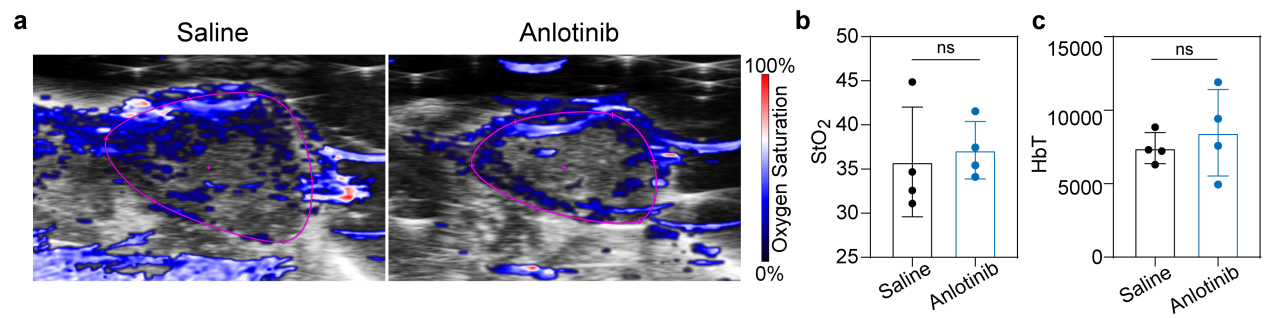


Fig. S13 Photoacoustic imaging of tumors from mice treated with saline or anlotinib for 10 days. **(a)** Ultrasound image in grayscale overlaid with Oxygen saturation (StO_2_) map of tumors. **(b)** Mean StO_2_ values (n = 4). **(c)** Mean total hemoglobin (HbT) values (n = 4). The data are shown as the mean ± s.d.


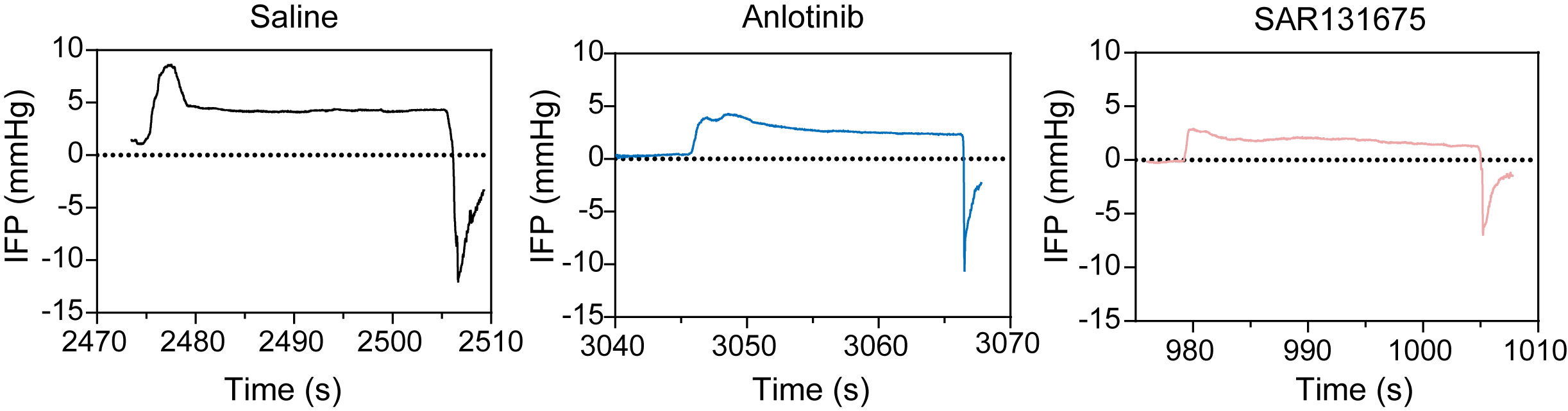


Fig. S14 Representative tumor IFP curves from different treatment groups.


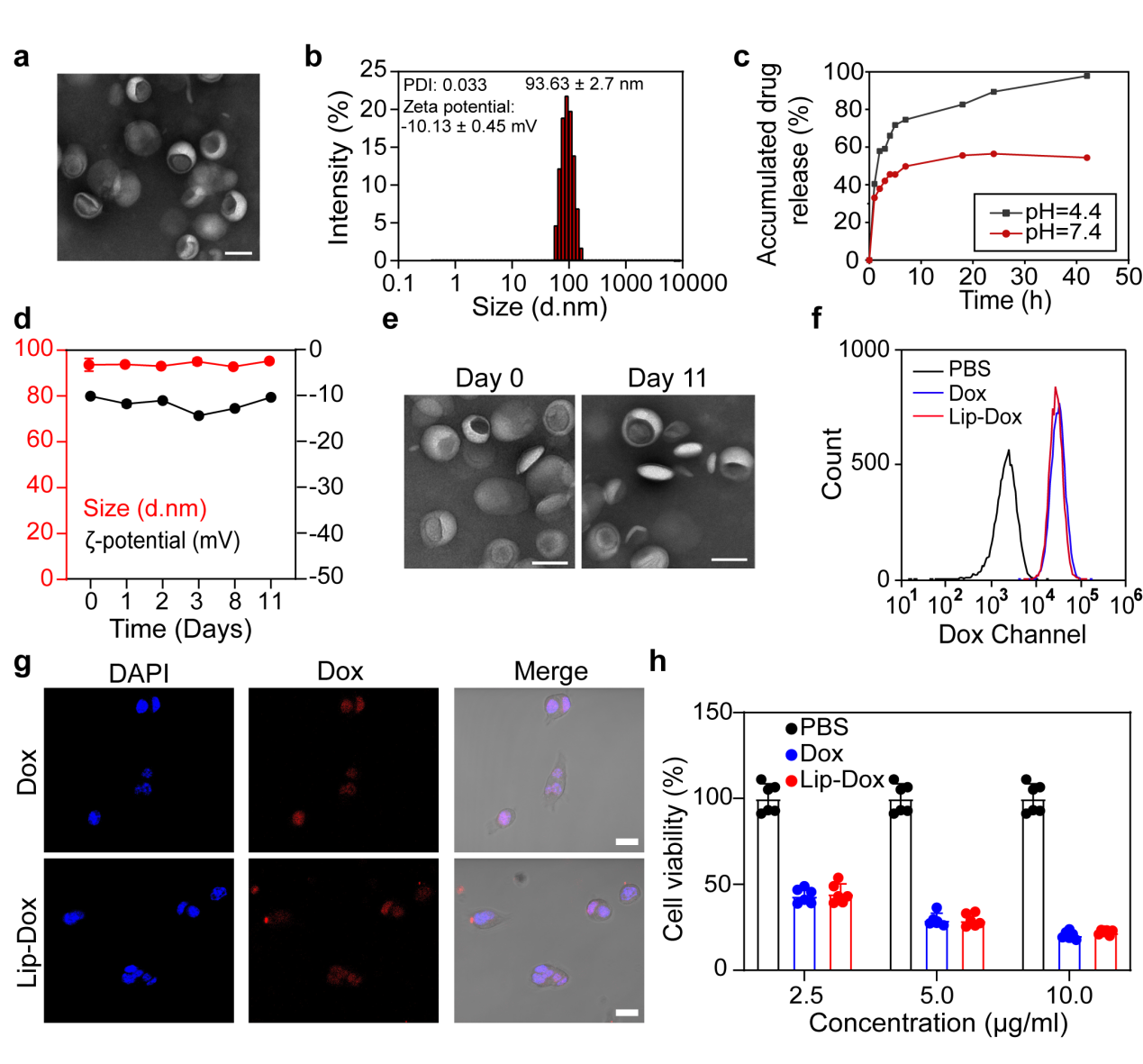


Fig. S15 Characterization of Lip-Dox. **(a)** TEM image of Lip-Dox. Scale bar, 100 nm. **(b)** Size distribution and Zeta-potential of Lip-Dox, as determined by DLS (n = 3). **(c)** Release kinetics of Dox from Lip-Dox at pH 7.4 and pH 4.4, as measured by UV‒Vis spectrophotometry. **(d-e)** Stability of Lip-Dox in PBS, as evaluated by changes in size distribution, zeta potential (d), and morphology (e) at the indicated time points (n = 3). Scale bar, 100 nm. **(f)** Flow cytometry analysis of 4T1 cells after incubation with Lip-Dox or Dox for 2 h. **(g)** Confocal laser scanning microscopy images of 4T1 cells after incubation with Lip-Dox or Dox for 2 h. **(h)** Cytotoxicity of Lip-Dox against 4T1 cells (n=6). The data are shown as the mean ± s.d.


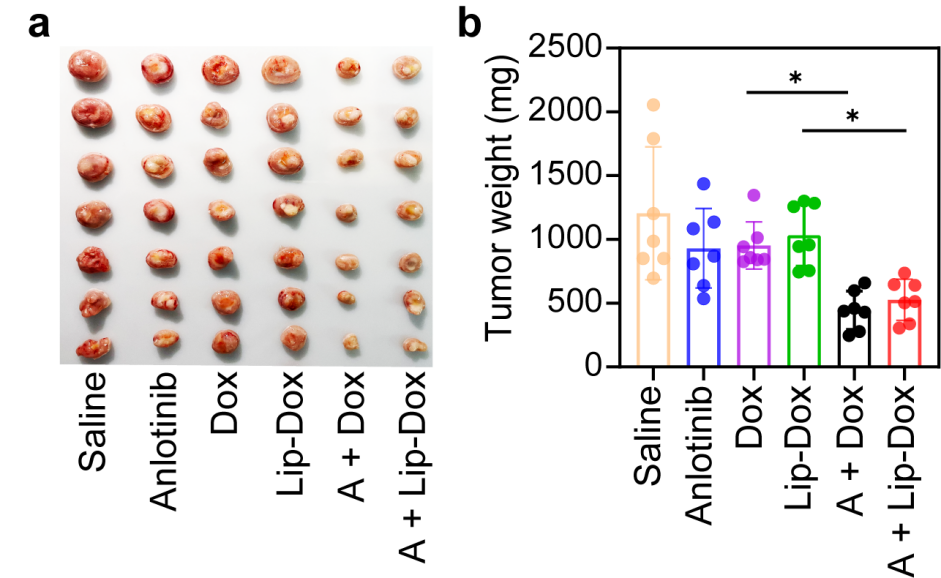


Fig. S16 **(a)** Image of excised CT26 tumors. **(b)** Tumor weights of the CT26 tumor model after treatment (n = 7). The data are shown as the mean ± s.d. **p* < 0.05.


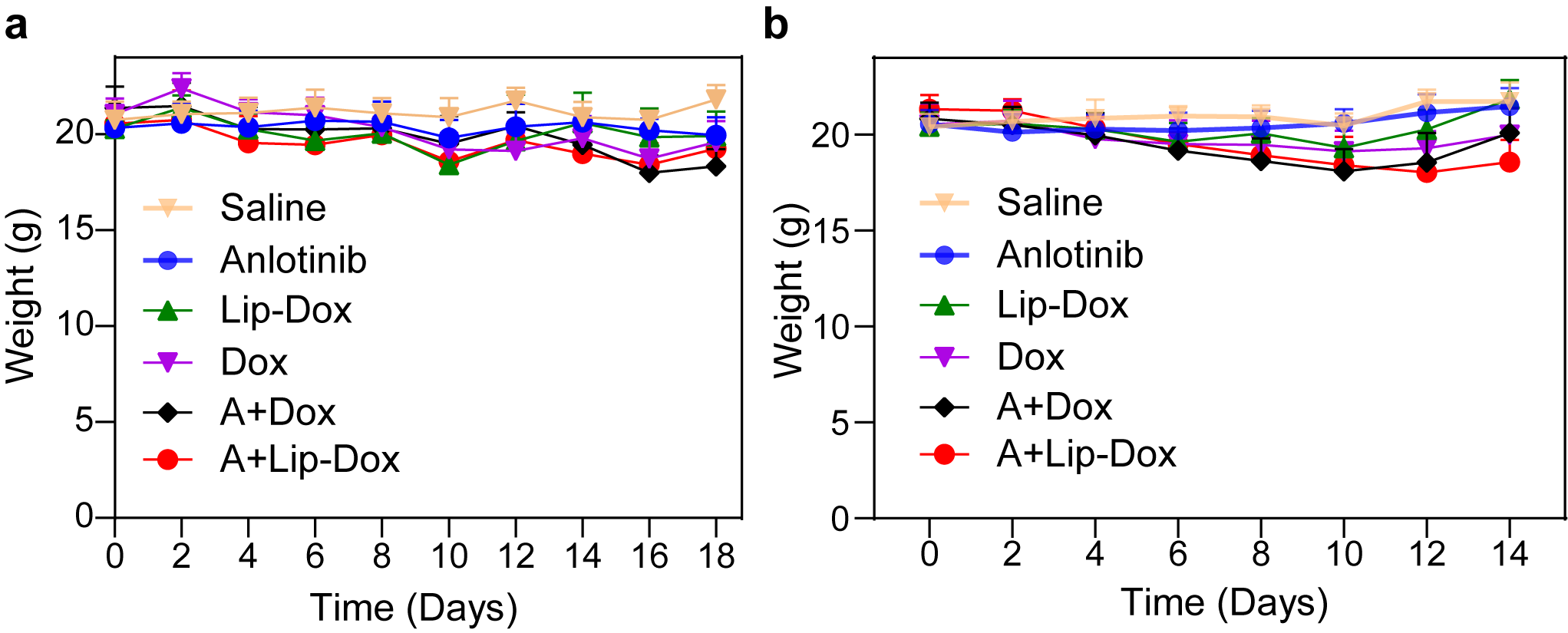


Fig. S17 Body weight changes during combined therapy. **(a)** Changes in the body weight of 4T1 tumor-bearing mice over the 18 days of treatment (n = 5). **(b)** Changes in the body weight of CT26 tumor-bearing mice during the treatment period (n = 7). The data are presented as the mean ± s.d.


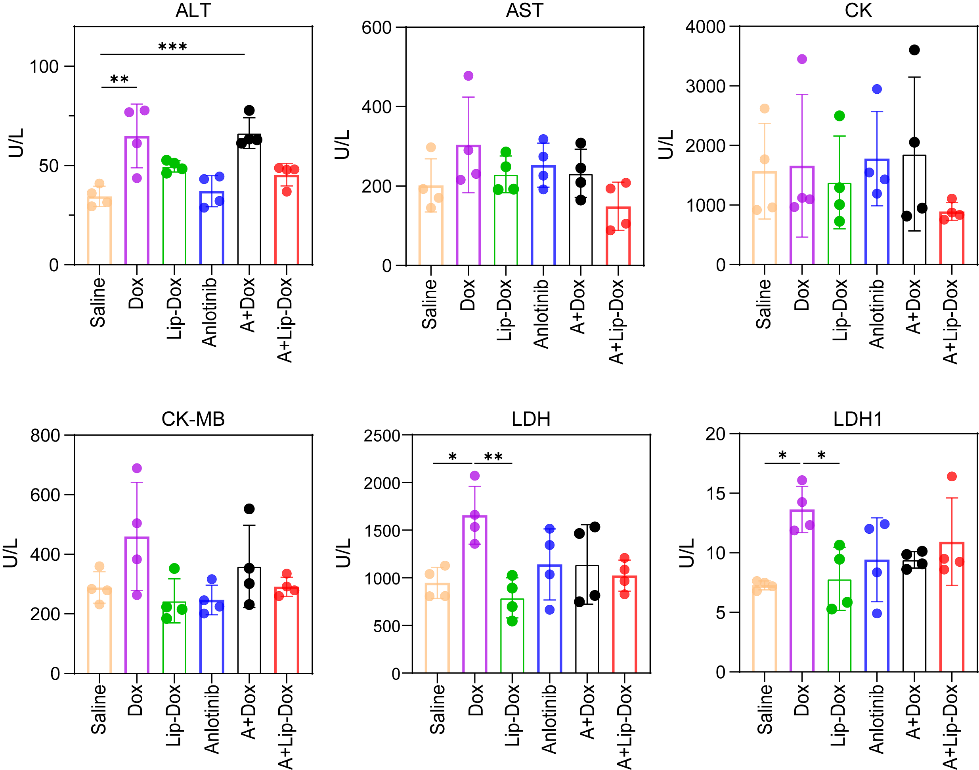


Fig. S18 Serum biochemistry analysis of CT26 tumor bearing mice after the indicated treatments. ALT, alanine aminotransferase; AST, aspartate aminotransferase; CK, creatine kinase; CK-MB, creatinine kinase isoenzyme MB; LDH, lactate dehydrogenase; LDH1, lactate dehydrogenase isoenzyme 1 (n = 4). The data are presented as the mean ± s.d. **p* < 0.05, ***p* < 0.01, ****p* < 0.001.


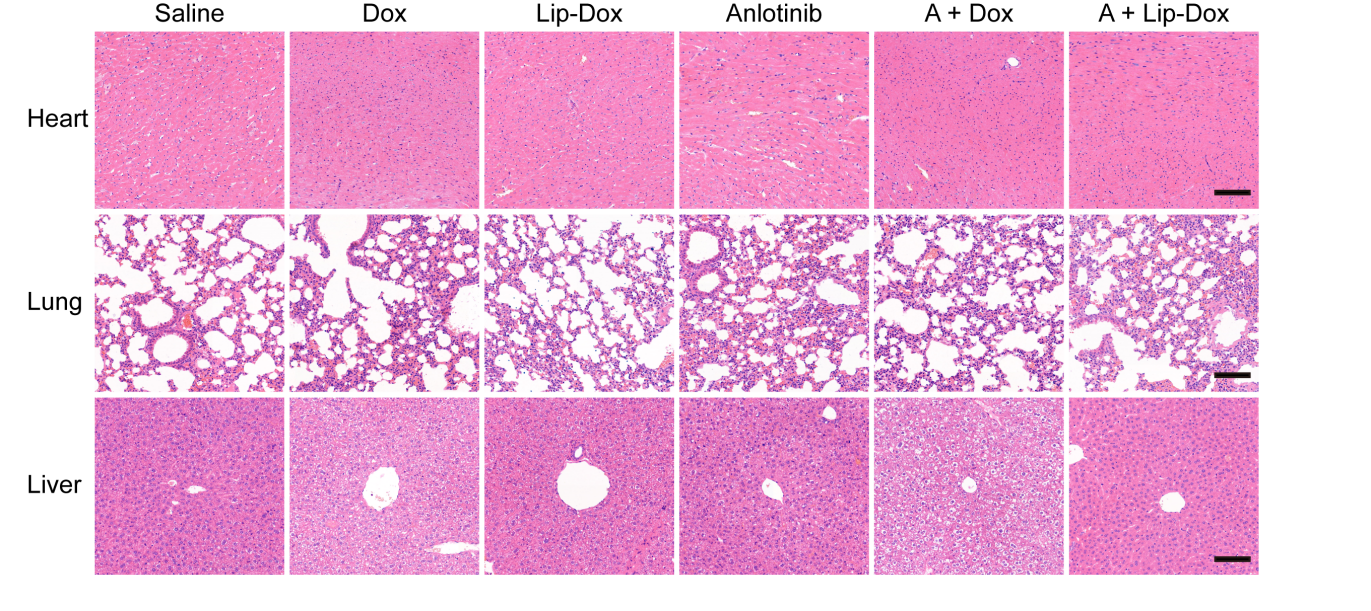


Fig. S19 H&E staining of the major organs from CT26 tumor bearing mice. No apparent morphological changes were observed after the indicated treatments. Scale bars, 100 μm.


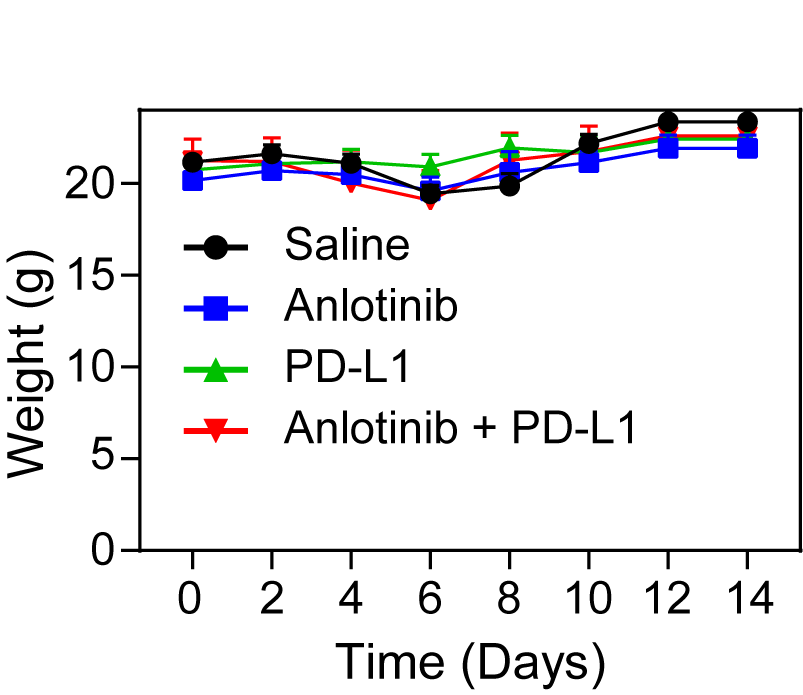


Fig. S20 Body weight changes in mice after the indicated treatments. Neither the anti-PD-L1 antibody nor anlotinib caused significant body weight loss (n = 6). The data are presented as the mean ± s.d.


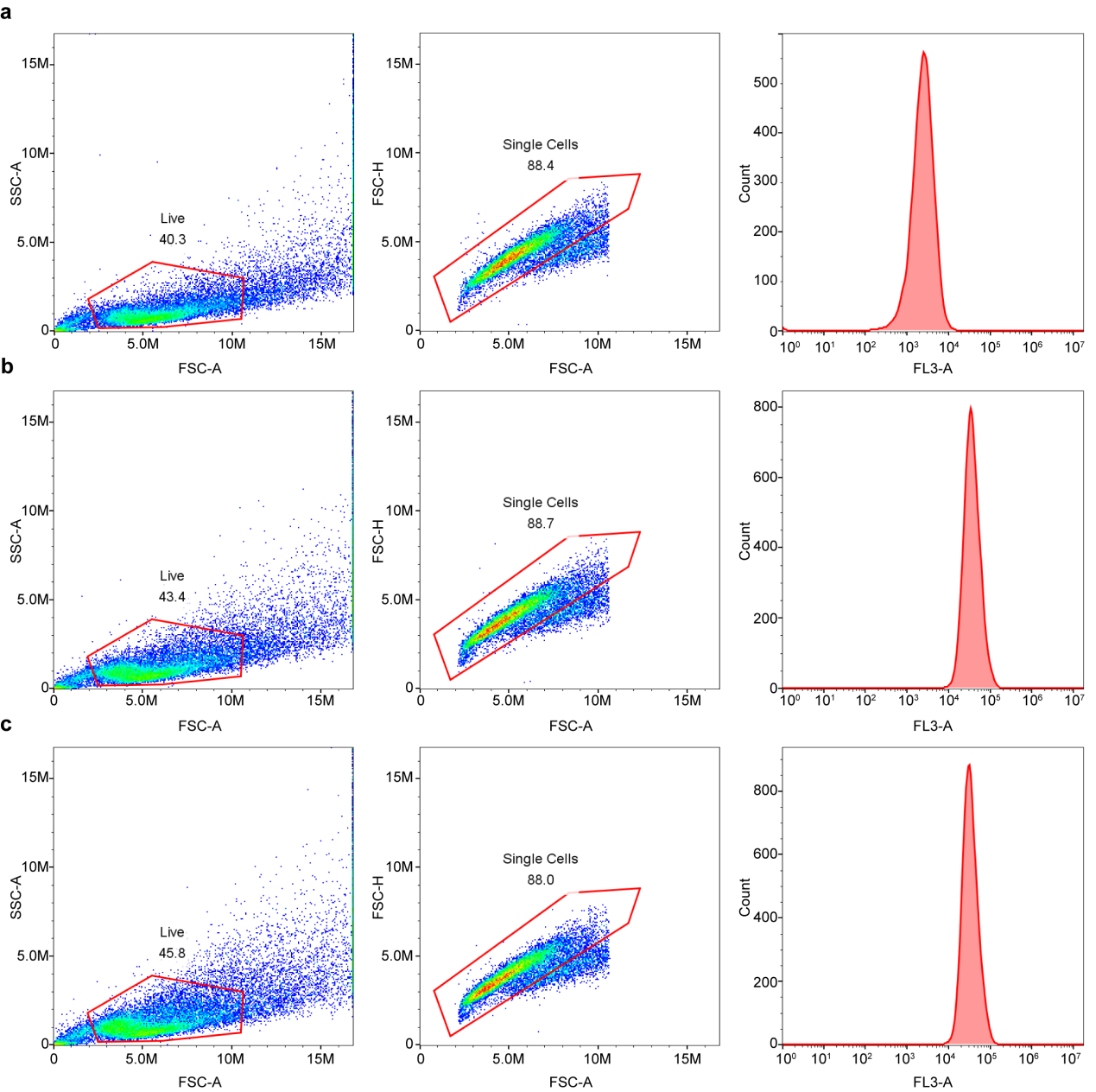


Fig. S21 Gating strategy for Figure S15f. **(a)** Untreated 4T1 cells. **(b)** Dox-treated 4T1 cells. **(c)** Lip-Dox-treated 4T1 cells.

Fig. S22 Original and uncropped films of Western blots.


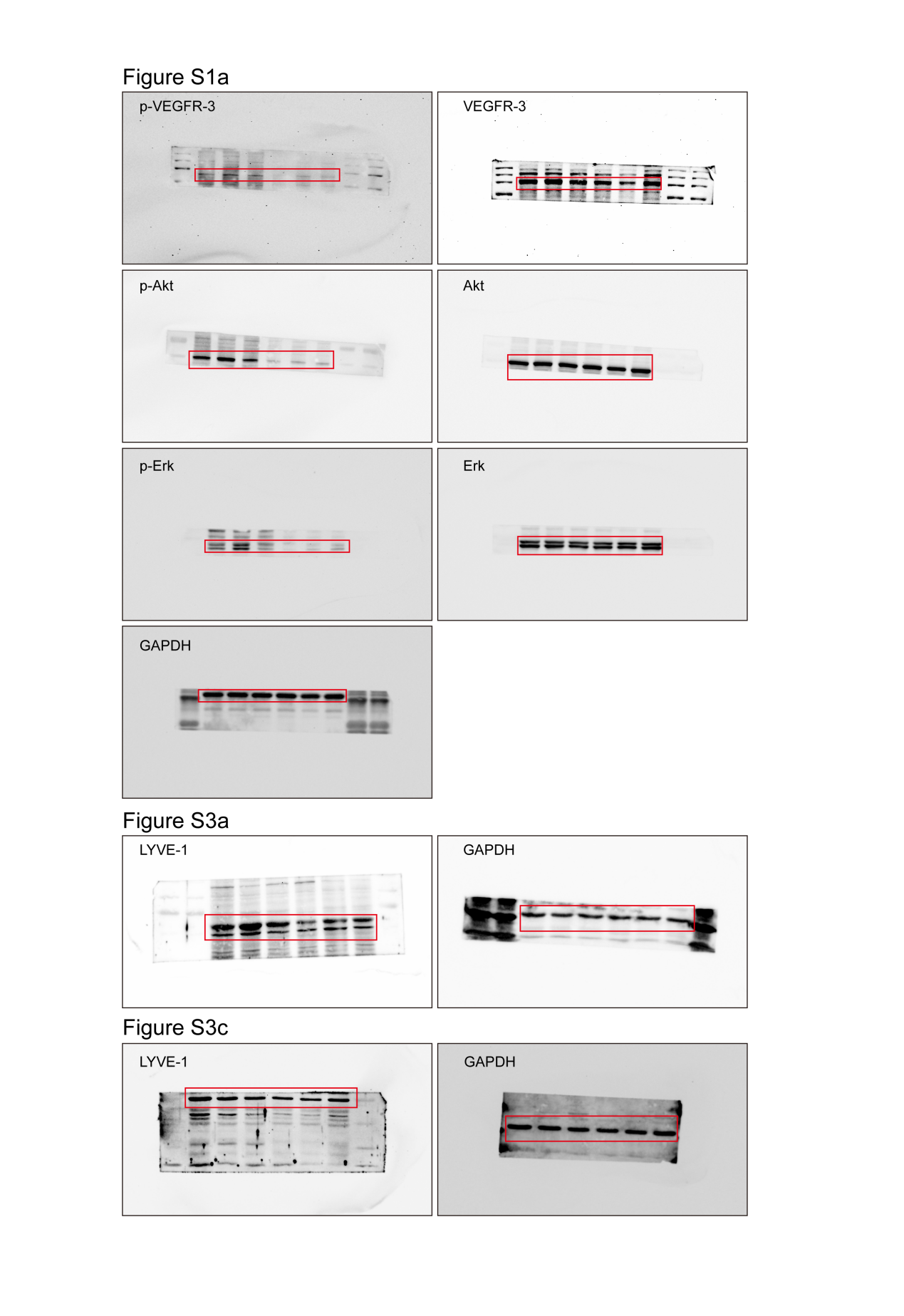

Supplement: Supplementary file 1 — Supporting Information [file 41392_2024_1794_MOESM1_ESM.docx]
